# Molecular Phylogeography of a Human Autosomal Skin Color Locus Under Natural Selection

**DOI:** 10.1534/g3.113.007484

**Published:** 2013-11-01

**Authors:** Victor A. Canfield, Arthur Berg, Steven Peckins, Steven M. Wentzel, Khai Chung Ang, Stephen Oppenheimer, Keith C. Cheng

**Affiliations:** * Department of Pharmacology, Penn State College of Medicine, Hershey, Pennsylvania 17033; † Penn State Cancer Institute, Penn State College of Medicine, Hershey, Pennsylvania 17033; ‡ Department of Public Health Sciences, Penn State College of Medicine, Hershey, Pennsylvania 17033; § Jake Gittlen Cancer Research Foundation, Penn State College of Medicine, Hershey, Pennsylvania 17033; ** Division of Experimental Pathology, Penn State College of Medicine, Hershey, Pennsylvania 17033; †† School of Anthropology and Museum Ethnography, Oxford University, Oxford, OX2 6PE, UK

**Keywords:** natural selection, skin color, *SLC24A5*, haplotype, recombination

## Abstract

Divergent natural selection caused by differences in solar exposure has resulted in distinctive variations in skin color between human populations. The derived light skin color allele of the *SLC24A5* gene, *A111T*, predominates in populations of Western Eurasian ancestry. To gain insight into when and where this mutation arose, we defined common haplotypes in the genomic region around *SLC24A5* across diverse human populations and deduced phylogenetic relationships between them. Virtually all chromosomes carrying the *A111T* allele share a single 78-kb haplotype that we call C11, indicating that all instances of this mutation in human populations share a common origin. The C11 haplotype was most likely created by a crossover between two haplotypes, followed by the *A111T* mutation. The two parental precursor haplotypes are found from East Asia to the Americas but are nearly absent in Africa. The distributions of C11 and its parental haplotypes make it most likely that these two last steps occurred between the Middle East and the Indian subcontinent, with the *A111T* mutation occurring after the split between the ancestors of Europeans and East Asians.

Human skin pigmentation varies widely between populations, generally decreasing with distance from the equator. According to a hypothesis proposed by Loomis (1967) and elaborated by Jablonski and Chaplin (2000), decreased exposure to solar ultraviolet at high latitudes produces a strong selective advantage for decreased skin pigmentation because it permits increased dermal vitamin D synthesis. Consistent with this hypothesis, in people of European descent, the pigmentation locus *SLC24A5* shows strong evidence of selection (Lamason *et al.* 2005; Sabeti *et al.* 2007; Grossman *et al.* 2010) A specific coding polymorphism in this gene (rs1426654) is a major contributor to the pigmentation difference between Africans and Europeans (Lamason *et al.* 2005; Stokowski *et al.* 2007). Frequencies display strong population differentiation, with the derived light skin pigmentation allele (*A111T*) fixed or nearly so in all European populations and the ancestral allele predominant in sub-Saharan Africa and East Asia (Lamason *et al.* 2005; Norton *et al.* 2007). The genomic region of diminished sequence variation in Europeans spans ~150 kb (Lamason *et al.* 2005). To learn about the time and location of origin of the *A111T* mutation, we studied haplotypes in the region around *SLC24A5* across world populations.

## Materials and Methods

**Figure 1:**
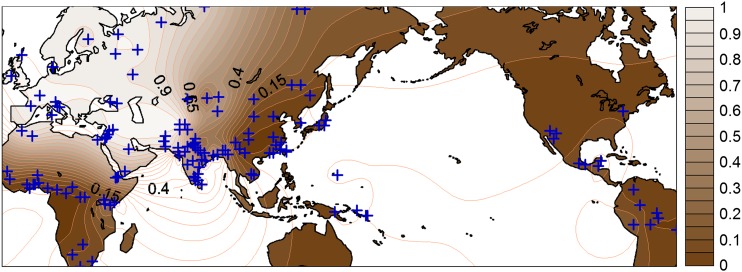
World distribution of *A111T* polymorphism in *SLC24A5*. The origins of sampled populations are indicated (+). Contours of global frequencies of *SLC24A5^A111T^* are shaded according to the frequency/color scale to the right. Frequency data by population that were used for this figure are tabulated in Table S1.

Population-specific frequency data used for *A111T*, shown in Figure 1 and Supporting Information, Table S1, were obtained from the following sources: HapMap populations (Altshuler *et al.* 2010) (http://hapmap.ncbi.nlm.nih.gov/); Human Genome Diversity Project (HGDP) populations (Norton *et al.* 2007) or the CEPH database (http://www.cephb.fr/en/cephdb/); populations from Sri Lanka (Soejima and Koda 2007); ALFRED (Cheung *et al.* 2000) (alfred.med.yale.edu/alfred/); Indian samples (Indian Genome Variation Consortium 2008) (http://igvdb.res.in); and additional Mediterranean and South Indian samples (Behar *et al.* 2010). HapMap populations consist of CEU (CEPH individuals of northern and western European descent, sampled in Utah), TSI (Tuscan, Italy), GIH (Gujarati, sampled in Houston), CHB (Chinese, Beijing), CHD (Chinese descent, sampled in Denver), JPT (Japanese, Tokyo), YRI (Yoruba, Ibadan, Nigeria), LWK (Luhya, Webuye, Kenya), MKK (Maasai, Kinyawa, Kenya), ASW (African-Americans from southwest United States), and MEX (Mexican, sampled in Los Angeles).

Haplotypes were derived from several datasets. Haplotypes initially were classified by use of the polymorphism data of HapMap phase 3 (Altshuler *et al.* 2010). Denser data such as those of the 1000 Genomes Project phase 1 data (1000 Genomes Project Consortium 2012) were used to define phylogenetic relationships between haplotypes. Clarity also was gained by considering haplotypes within blocks of linkage disequilibrium, designated A, B, C, and D, and naming longer haplotypes as combinations of these local haplotypes. Blocks A, B, and C+D are delimited by locations of recombination hotspots (estimated rates >5 cM/Mb) deduced from HapMap phase 2 data (International HapMap Consortium *et al.* 2007), whereas the boundary between blocks C and D was defined by an interval with modestly elevated recombination (~0.8 cM/Mb). Precise boundaries chosen were nt 48321236−48370103 for A, 48370104−48390052 for B, 48390053−48468019 for C, and 48468020−48517471 for D, using b37/hg19 coordinates.

Initial analysis of regions A and B used only single-nucleotide polymorphisms (SNPs) from HapMap Phase 3, Release 27 (Altshuler *et al.* 2010) that had been genotyped and phased in each included population (11 and 6 SNPs, respectively), whereas for regions C and D, SNPs lacking data for the TSI and/or MEX samples also were included (16 and 8 SNPs, respectively); these SNPs are listed in Table S2. Phased haplotypes were retrieved from HapMap and compared with raw genotype data for consistency. For the core region C, the absence of genotypes for SNPs c1 and c13 prevented distinguishing between members of the C2/C3 haplotype pair (in TSI) and the C6/C7 pair (in TSI and MEX), respectively. For TSI and GIH, which were phased on the basis of CEU, a small number of individuals heterozygous for the ancestral allele of *A111T* (1/1 and 3/8, respectively) were misphased. Corrected haplotype assignments are shown in all figures and tables except Figure 5 and those in which haplotype combinations are displayed (Figure S2, Figure S3, File S2, File S3, and File S4). For subregion D, only 1 of 8 SNPs was genotyped in TSI, precluding haplotype assignment for this sample.

A more complete picture of variation was obtained by examining phased haplotype information from the 1000 Genomes Project (http://mathgen.stats.ox.ac.uk/impute/ALL_1000G_phase1integrated_v3_impute.tgz; phase 1 version 3 of March 2012). The core region encompassed 767 polymorphisms in the core region, consisting of 23 biallelic indels and 744 SNPs. Because phasing becomes less reliable as allele frequency decreases and is undetermined for variants observed only once (287), analysis focused on the 156 polymorphisms with minor allele frequencies of 1% or greater, corresponding to 291 distinguishable haplotypes. A second dense set of SNPs also was compared with HapMap phase 2 data. After removal of monomorphic positions, 84 SNPs remained, 18 of which were found only in rare haplotypes. Phased haplotypes were retrieved from HapMap, Release 21. For phylogenetic analysis, graphs were drawn by the use of a simple nearest-neighbor approach and rooted by the use of ancestral alleles determined by comparison with other primate sequences. The few recombination events were identified by inspection, appearing as polymorphisms with transitions on more than one edge.

For HGDP sample haplotypes, the data of Li *et al.* (2008) were used. The genotype data contain 13 SNPs in region C that include 8 SNPs shared with HapMap Phase 3 plus 5 additional SNPs listed in Table S2. Phased haplotypes were estimated using Phase 2.1.1 (Stephens *et al.* 2001; Stephens and Donnelly 2003). In this dataset, all haplotypes were distinguishable except C6/C7 and C9/C10, due to the absence of data for SNP c13 for the former and c2 or c12 for the latter. Although the dataset lacks genotypes for SNP c11 (rs1426654) (Li *et al.* 2008), two independently obtained genotypes (Norton *et al.* 2007) and http://www.cephb.fr/en/hgdp/main.php (laboratory HDCEPH68) were available. These were generally consistent with the deduced haplotypes (~1% mismatch) and were used to distinguish two haplotype pairs that were not otherwise distinguishable (C2 and C11 *vs.* C3 and C9/C10). Similar procedures were used to analyze haplotypes for seven Middle Eastern, North African, East African, and South Indian samples by the use of the data from Behar *et al.* (2010). The genotype data contain the same set of SNPs available for the HGDP, plus SNP c11 and rs4775737.

Because haplotypes were derived from a variety of datasets, we tested both SNPs and deduced haplotypes for consistency. In the genomic region analyzed, we found no evidence of errors in allele designation. No source reported common haplotypes that did not correspond to those found in other datasets. All common core haplotypes in HapMap Phase 3 samples were represented by unambiguously phased examples. For a small number of individuals, HapMap and 1000 Genomes analyses yielded divergent phasing across the B-C region boundary. We have avoided drawing conclusions on the basis of rare haplotypes that could be artifactual products of genotyping or phasing errors.

To estimate the age of the *A111T* mutation, we used a molecular clock approach. We first determined the rate of mutation in the combined C and D regions from number of differences between human and chimpanzee reference sequences. In this alignment, nt changes within 4 nt of gaps were excluded to remove potential biases caused by misalignment. For calibration, we used 6 million years, the midpoint of the range of estimates (5−7 million years) for the divergence time between human and chimpanzee as identified by Kumar *et al.* (2005) and assumed equal mutation rates (per year) in the human and chimpanzee lineages. We then counted the number of single-nucleotide differences from the modal haplotype for each C11-D4−containing chromosome in the 1000 Genomes dataset by using all reported variants. Each chromosome provides an imprecise estimate of the time since the origin of the haplotype; values were averaged over individual populations, or the entire sample. Because accumulation of mutations in a single lineage is independent of population size, this procedure does not require demographic assumptions or data. Although *A111T* is subject to selection, we assume only that subsequent mutations are neutral. Our approach to dating selective sweeps differs from that used by Rozas *et al.* (2001) and Meiklejohn *et al.* (2004), which counts the number of affected sites and underestimates the coalescence time unless each sampled lineage is independent. In contrast, our estimate is unbiased in the presence of nonindependent samples. Because the most frequent C11 + D4 variant carrying an additional mutation occurs 36 times in 1013 chromosomes, the independence condition is clearly not met.

To estimate confidence limits, we considered the effective sample size to be the number of chromosomes that could be sampled without violating the assumption of independence. This was determined by resampling, with replacement, until a variant haplotype was duplicated. The median counts without repeat in 10,000 replicates ranged from 9 to 14 for individual population subsamples, to 19 for the combined sample, substantially lower than the total number of chromosomes sequenced. These values, multiplied by the observed mutation frequencies, were then used to calculate confidence limits under the assumption that mutations follow a Poisson distribution.

The aforementioned approach is most applicable when all variants in the chromosomal region under study have been determined. At the current sequencing depth in 1000 Genomes data (5.1-fold across autosomes), we expect some rare variants to be undetected, resulting in an underestimate of the age of *A111T*. We made an approximate correction for the underestimate by inverting the estimated power relationship (1000 Genomes Project Consortium 2012). In particular, the corrected count is given by

$$\Sigma i\left(A_{i}/P_{i}\right)$$

where *A_i_* is the observed number of sites at which variants are reported to occur *i* times in the sample and *P_i_* is the power to detect variant sites with *i* occurrences. This calculation ignores the effects of miscounts (*e.g.*, a variant occurring three times is detected but inaccurately reported as having two or four occurrences), and assumes that the genome-wide power applies to the genomic region under investigation. Because the largest contribution is undetected singletons (87% of the estimated excess), the assumption that miscounts contribute little to the outcome is reasonable. Applying this procedure using the SNP frequency spectrum for C11-D4 across all samples suggests that the reported age range should be increased by a factor of approximately 1.58.

Comparing different population subsamples, we noted a trend toward greater counts in the admixed New World populations. Misassignment of rare or unique variants in individuals heterozygous for *A111T* (common in the admixed, but rare in the European samples) was a potential explanation of this trend, but the absence of any difference in variant frequency between heterozygotes and homozygotes in pooled Puerto Rican/ Colombian/Mexican samples suggests that this was not a significant source of bias.

## Results and Discussion

### Global distribution of the *A111T* mutation in *SLC24A5*

The geographical distribution of the *A111T* allele of *SLC24A5* (Norton *et al.* 2007), updated with the use of additional population samples (Figure 1), shows that *A111T* is nearly fixed in all of Europe and most of the Middle East, extending east to some populations in present-day Pakistan and north India. *A111T* shows a latitudinal decline toward the Equator, with high frequencies in Northern Africa (>0.80), intermediate (0.4−0.6) in Ethiopia and Somalia, and lower (<0.35) in sub-Saharan Africa. This pattern is broadly consistent with strong positive selection for decreased skin pigmentation throughout Europe. There is a cline of decreasing frequency of *A111T* in indigenous populations east of approximately longitude 75° in Central Asia, with near-absence in East Asia, Oceania, and the Americas. The extent to which the spread of *A111T* to the east has been inhibited by the absence of substantial eastward population migrations postdating its origin or by the presence of other loci responsible for decreased skin pigmentation in East Asia is presently unclear.

### Characterization of haplotypes in the genomic region encompassing *SLC24A5*

**Figure 2:**
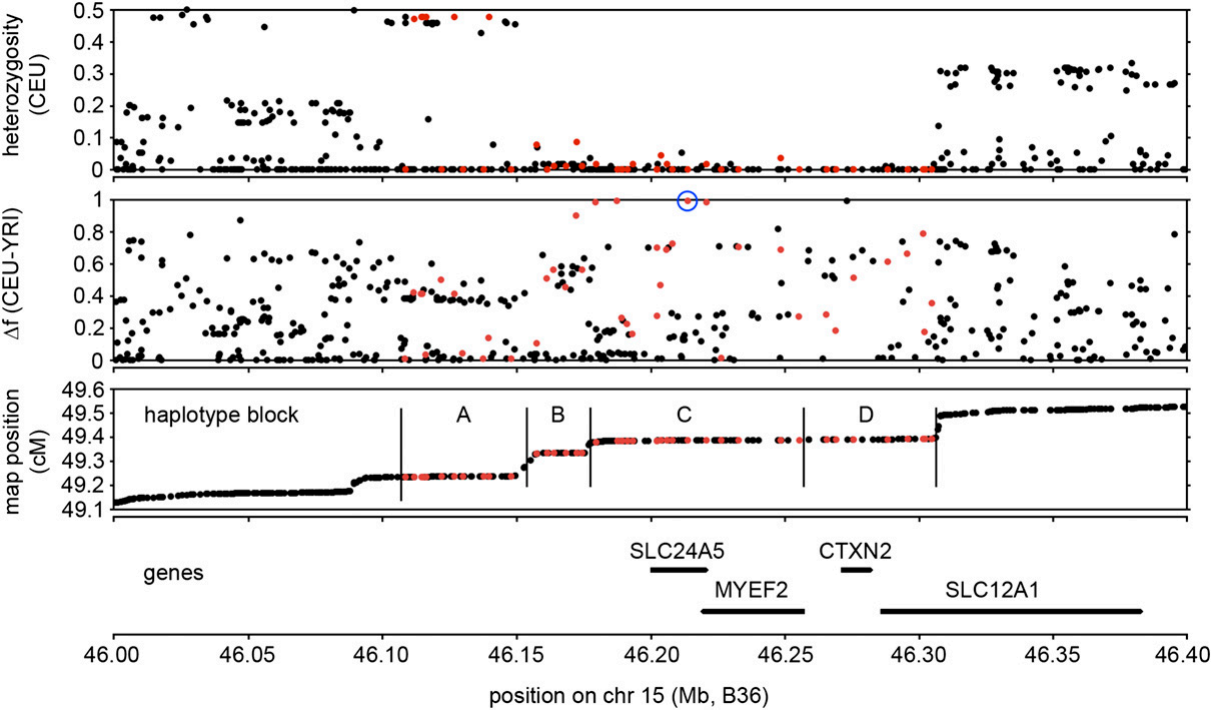
Genomic region surrounding *SLC24A5*. The first three panels show SNPs (red symbols, SNPs used for haplotype analysis in HapMap Phase 3, Release 27, also listed in Table S2; black symbols, additional SNPs genotyped in fewer HapMap populations, or flanking SNPs not analyzed here). SNPs monomorphic in the original four HapMap population samples (CEU, CHB, JPT, YRI) are omitted. Top, SNP heterozygosity in CEU, calculated from allele frequencies, illustrating the region of diminished variation in this European sample. Middle, SNP allele frequency difference between CEU and YRI, showing several SNPs with extremely high frequency differentiation (Δf ≥ 0.8); rs1426654 is circled. Beneath is a recombination map showing boundaries of blocks used for haplotype analysis (A through D). Region C, as described in text, is the core region containing *SLC24A5*. Bottom, positions of *SLC24A5* and other genes in region, with common coordinates (NCBI build B36) used for all panels. The centromere is to left.

**Figure 3:**
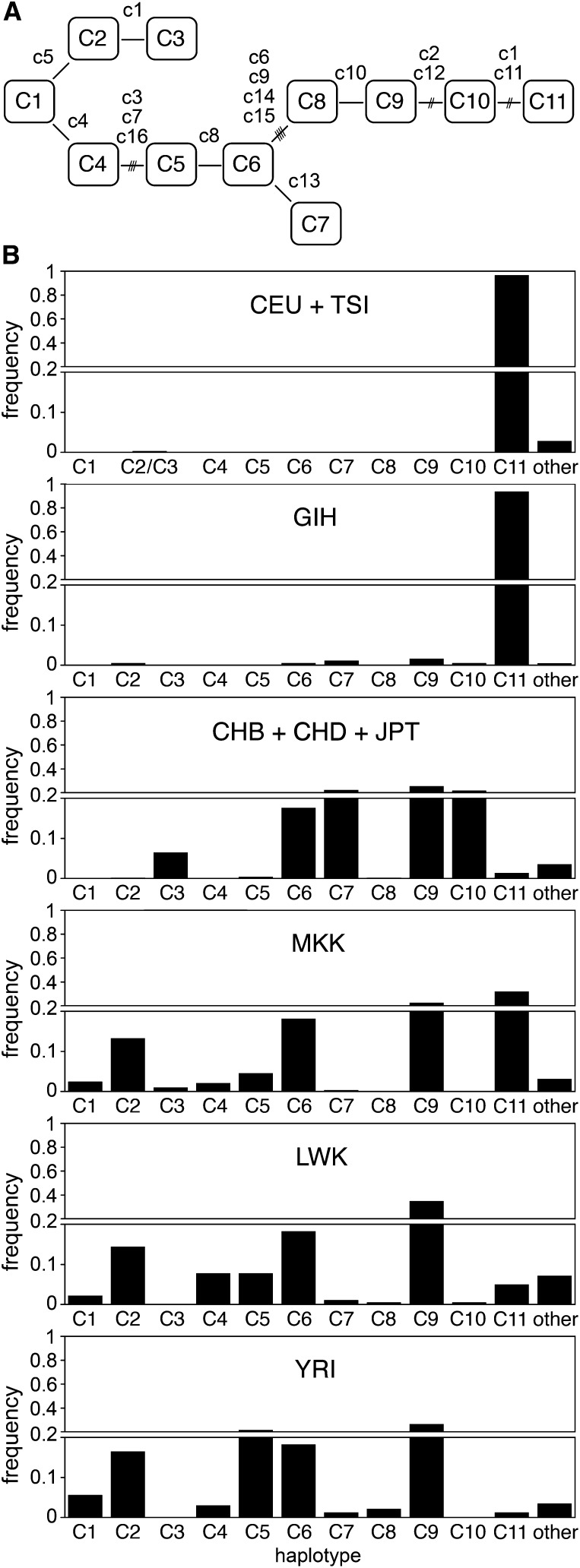
Core-region haplotypes defined using 16 SNPs. (A) Diagram showing relationships between common haplotypes C1 through C11. SNPs that differ between adjacent haplotypes are labeled by nickname (SNP c1 through c16, keyed to Table S2). Haplotype C1 contains ancestral alleles for each SNP in this series. Haplotype C11 carries the derived Thr-111 allele of *SLC24A5* (rs1426654, SNP c11). The only duplicated transition in this diagram is for SNP c1 (rs1834640). Note that analysis of additional SNPs reveals cases in which adjacent haplotypes in the diagram do not have ancestor-descendent relationships (compare Figure 4). (B) Histograms showing the distribution of haplotypes in several HapMap populations. The three East Asian populations are combined, as are CEU and TSI. Indeterminate C2/C3 in TSI indicated. MEX and ASW are not depicted. The frequency scale is split at 0.2.

**Figure 4:**
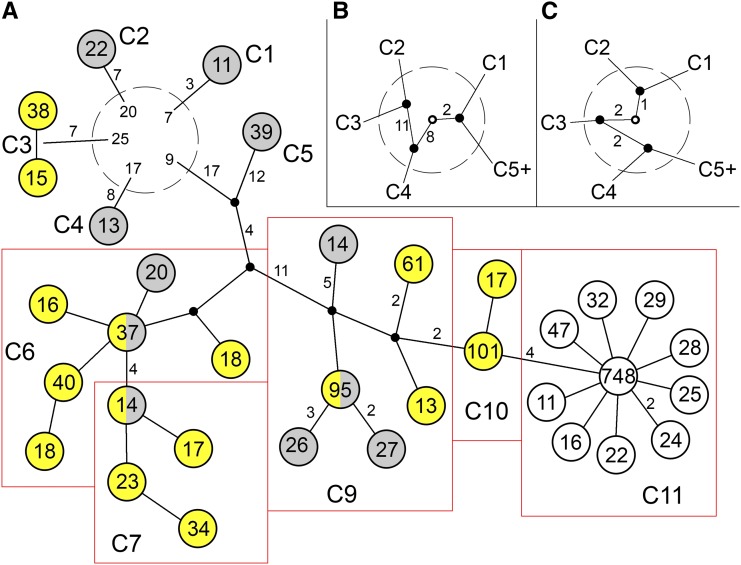
Phylogenetic relationships among core haplotype clusters. Haplotypes were deduced from 1000 Genomes data by the use of 156 polymorphisms with frequency ≥1%. (A) Haplotypes having frequencies ≥0.5% (representing 78% of total) are depicted. Complete dataset is found in File S1. Sets of haplotypes corresponding to those deduced using 16 SNPs are enclosed within red boxes and labeled C1 through C11. C8, which constituted <0.5%, is not shown. Two variants of C3 differ at an indel that shows evidence of recurrent mutation and for which the ancestral state is unknown. Values within each circle indicate number of occurrences. Gray and yellow shading indicates haplotypes that are predominantly African or East Asian (in this dataset), respectively. Numbers of polymorphic differences on each branch are indicated if greater than one. Inclusion of lower frequency polymorphisms would raise the number of C1-specific variants to 26, whereas C4-specific variants would increase to 11. Early haplotype lineage divergence, depicted within the dashed circle, provides evidence for multiple recombination events that are not resolved in this phylogeny. The numbers within the dashed circle represent number of polymorphic differences from the ancestral state; this involves 31 polymorphisms that have derived alleles shared by more than one lineage. (B) Relationships among early diverging branches supported by 21 polymorphisms. (C) Alternative relationships among early diverging branches supported by 5 polymorphisms. The ancestral state is represented by the open circle in B and C.

Diminished variation in the genomic region around *SLC24A5* in the HapMap CEU (European ancestry) sample led us to ask what the haplotypes associated with the *A111T* allele looked like, and how, when, and where they might have arisen. We therefore investigated haplotypes spanning this genomic region (Figure 2). The haplotypes are described in the context of four contiguous subregions defined by blocks of linkage disequilibrium, here designated A (49 kb), B (20 kb), C (78 kb), and D (49 kb) (Figure 2). Blocks B, C, and D together encompass the region of diminished variation in CEU. Analysis of the core subregion C, which includes *SLC24A5*, yielded 46 haplotypes in HapMap Phase 3 populations (Table S3 and Table S4). The 11 haplotypes with individual abundances >0.5%, which we designate C1 through C11, collectively comprise 93–98% of the total in each population (Figure 3, Table 1, and Table 2). A single haplotype, C11, accounts for 97% of all instances of the *A111T* variant of *SLC24A5*. Most of the haplotypes with frequencies <0.5% appear to be products of recombination between more frequent haplotypes. Analysis of common haplotypes found in HGDP and other samples (Li *et al.* 2008; Behar *et al.* 2010) yielded results matching those derived from HapMap samples (Table S5 and Table S6), including the equivalence between haplotype C11 and the derived allele of rs1426654. This finding is consistent with a common origin for *A111T* worldwide. Analysis of data from the 1000 Genomes Project indicated that haplotypes defined on the basis of 16 SNPs corresponded to sets of closely related haplotypes (Figure 4 and File S1). Common haplotypes defined using 1000 Genomes data differed from the ancestral state at 27−48 positions. The number of haplotypes detected depends on the number of polymorphisms used to define them; with inclusion of lower frequency variants, an increasing fraction of chromosomes corresponds to rare haplotypes (Table S7).

**Table 1:** Description of common core-region haplotypes defined using 16 SNPs

Haplotype	Polymorphism															
SNP ID	rs1834640	rs2675345	rs2469592	rs2470101	rs938505	rs2433354	rs2459391	rs2433356	rs2675347	rs2675348	rs1426654	rs2470102	rs16960631	rs2675349	rs3817315	rs7163587
Nickname	c1	c2	c3	c4	c5	c6	c7	c8	c9	c10	c11	c12	c13	c14	c15	c16
Ancestral state	G	G	G	C	C	T	G	A	G	G	G	G	A	G	T	T
C1																
C2					T											
C3	A				T											
C4				T												
C5			A	T			A									C
C6			A	T			A	G								C
C7			A	T			A	G					G			C
C8			A	T		C	A	G	A					A	C	C
C9			A	T		C	A	G	A	A				A	C	C
C10		A	A	T		C	A	G	A	A		A		A	C	C
C11	A	A	A	T		C	A	G	A	A	A	A		A	C	C

Only differences from ancestral allele are shown. Rare core-region haplotypes (omitted here) are tabulated in Table S3.

**Table 2:** Distribution of common core-region haplotypes in HapMap populations

Haplotype	HapMap Population										
	CEU	TSI	GIH	CHB	CHD	JPT	MKK	LWK	YRI	ASW	MEX
C1							7	4	13	1	
C2		}1*^a^*	1	1			38	26	38	21	1
C3				14	14	5	3				9
C4							6	14	7	3	
C5				1		1	13	14	50	19	
C6			1	34	21	35	52	33	42	20	}7*^b^*
C7			2	40	39	36	1	2	3	1	
C8					1			1	5	3	
C9			3	36	44	51	65	63	61	27	15
C10			1	34	41	37		1			9
C11	109	173	167	3	3	1	92	9	3	22	60
Other-d	6	2	1	2	1	1	2	3			2
Other-a				3	6	5	7	10	8	9	1
total	115	176	176	168	170	172	286	180	230	126	104

Zeroes representing absent haplotypes are omitted. Other-d indicates minor haplotypes carrying derived allele of SNP c11 (rs1426654), whereas other-a indicates minor haplotypes carrying its ancestral allele. Populations are described in the *Materials and Methods*.

a Haplotypes C2 and C3 are not distinguished in TSI.

b C6 and C7 are not distinguished in MEX.

### Phylogenetic relationships between core haplotypes

Phylogenetic relationships between haplotypes determined using 1000 Genomes data (Figure 4) were equivalent to those deduced from HapMap phase 2 data. The branches comprising C1, C2, C3, C4, and C5-C11 are early diverging clades. The most abundant and extensive lineage includes three branches: C5, C6-C7, and C9-C11. Within the C9-C11 branch, the C9 cluster is ancestral to C10, but nearly all instances of C9 include additional polymorphisms that distinguish them from common ancestors with C10. In contrast, the most commonly observed C10 variant is ancestral to C11.

### Identification of a 147-kb founder haplotype containing *A111T*

**Figure 5:**
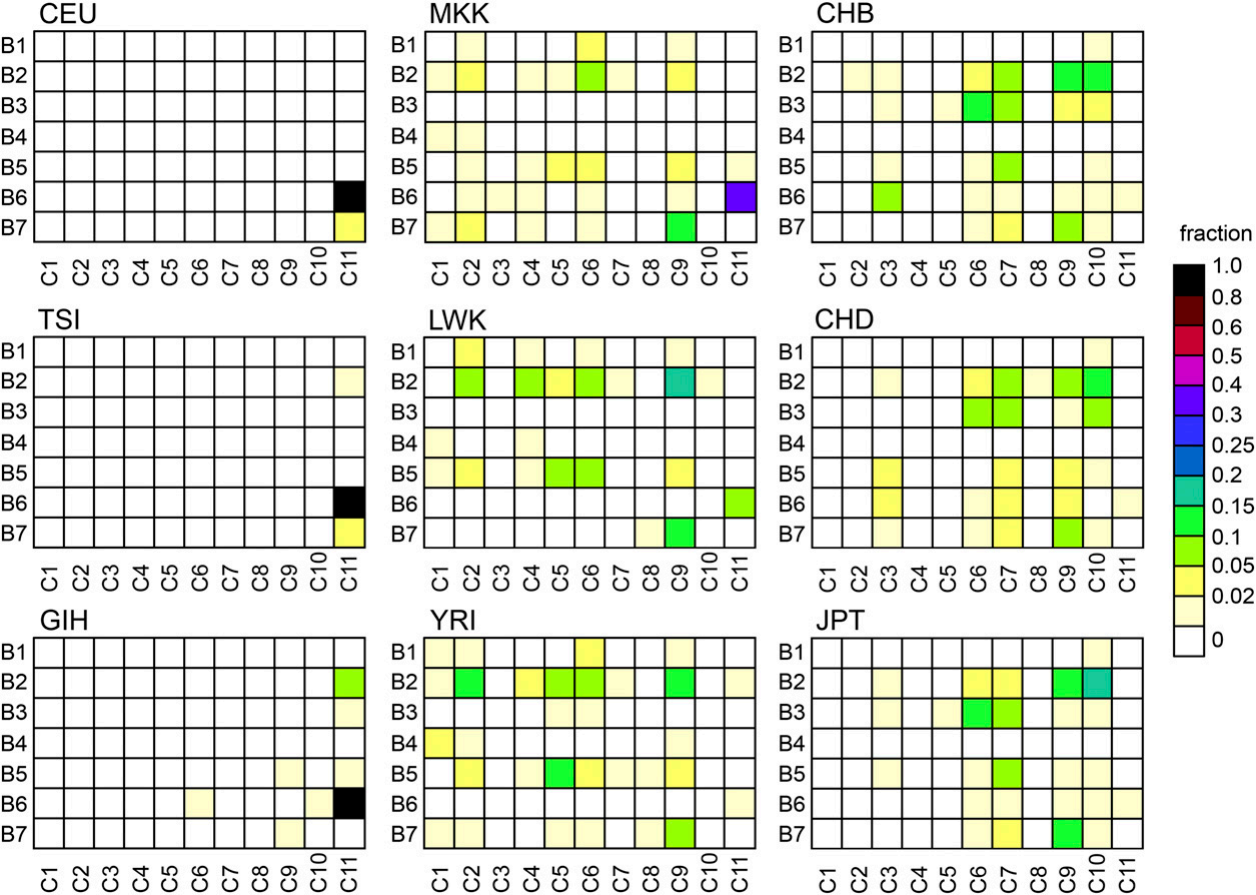
Relationships between local haplotypes in B and C regions. For each HapMap population, the distribution of haplotype combinations is shown as a heat map (scale on right). Recurrent recombination between core- and B-region is apparent. Predominant association of C11 with B6 contrasts with associations of C10 (B2) and C9 (B2, B7). Counts are shown in File S2.

The region of diminished variation in Europeans includes subregions B and D, flanking the core region (Figure 2). B- and D-region haplotypes are summarized in Figure S1, A and B and Table S8, Table S9, Table S10, and Table S11. It is apparent that a variety of combinations of B- and C-region haplotypes arose by recombination (Figure 5 and File S2). The smaller number of combinations of C- and D-region haplotypes observed (Figure S2 and File S3) is consistent with the occurrence of fewer crossovers near this location, including one ancestral to C10, C11, and a subset of C9. The B- and D-subregion haplotypes associated most strongly with haplotype C11 are B6 (94%) and D4 (>99%), respectively (Figure 5, Figure S2, and File S2 and File S3). This pattern also holds in HapMap populations in which C11 is far from fixation and in which a diversity of other B- and D-region haplotypes not associated with C11 are found, including MKK. Interestingly, the greatest diversity of B-region haplotypes associated with C11 is found in GIH (89% B6). Taken together, these results establish that the 147-kb founder haplotype containing *A111T* was B6 + C11 + D4.

Subregion A, to the left of subregion B, lies outside the region of diminished variation around *SLC24A5* observed in Europeans (Lamason *et al.* 2005), indicating that recombination occurred between the A and B regions after the origin of *A111T* but before its fixation. An analysis of the A subregion is shown in Figure S1C, Table S12, and Table S13. Two haplotypes, A1 and A5, predominate (together totaling 89–99%) in association with C11 (Figure S3 and File S4). The relative proportions of A1 and A5 associated with B6 + C11 vary considerably among populations (Table S14), presumably a result of genetic drift. The data do not allow a determination of whether A1 or A5 was a part of the founding *A111T* haplotype.

### Recombination was involved in the creation of C11

**Figure 6:**
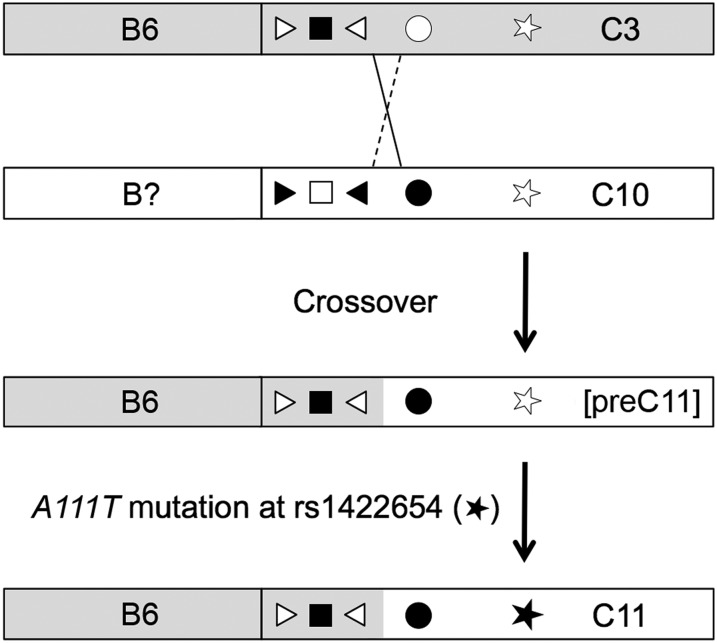
Generation of haplotype C11. Recombination between chromosomes containing C10 (white) and C3 (shaded) is indicated by X. The crossover product represented by the solid line is the precursor to C11; the reciprocal recombinant represented by the dotted line is not recovered. Ancestral alleles are represented by open symbols, and derived alleles are solid black, where rs12441154, rs1834640 (c1), rs2675345 (c2), rs57108441, and rs1426654 (c11) are represented by the triangle, square, triangle, circle, and star, respectively. The B6 haplotype (here defined by 6 SNPs), just to the left of the C region, is that most commonly found in phase with the C3 allele, as indicated; B2 and B3 are most commonly in phase with C10. Subsequent mutation at rs1426654, represented by the black star, produced the predominant *SLC24A5^A111T^*-containing haplotype, C11, which is globally associated with B6. 1000 Genomes Project data localize the crossover to the 0.9-kb interval between rs57108441 and rs78729596. Flanking regions are not shown. Note that the final product is identical under an alternate model in which mutation precedes recombination.

Haplotype C3 and C11 share the derived allele of SNP c1 (Table 1), suggesting the possibility of recombination. To test this possibility, we examined SNPs not genotyped in HapMap Phase 3. Haplotype C11 carries ancestral alleles of SNPs rs12441154 and rs57108441, whereas C10 carries the derived alleles, a pattern readily explained by a single crossover between C3 and C10 (Figure 6). In support of this notion, the B-region haplotype found associated with C11, B6, is also the one most commonly associated with C3; conversely 96% of C10 haplotypes are associated with B region haplotypes other than B6 (68% with B2; File S2).

Can we determine whether recombination involving C3 preceded or followed the mutation that created *A111T*? Models in which the recombination or mutation occurred first produce the same end product but proceed through different intermediates, corresponding to C26 or C22, respectively (Table S3). Rare haplotypes matching both potential C11 precursors were found. Because either could have been produced by recombination subsequent to the origin of C11, their occasional occurrence is not informative. However, an evolutionary argument strongly suggests an order of events (Figure S4). If the crossover predated the mutation, the predicted intermediate (C26) would not have experienced positive selection on the basis of lighter pigmentation (Figure S4A). Selection for decreased skin pigmentation would cause the predominant haplotype containing *A111T* to be C11, as is observed. On the other hand, if the *A111T* mutation preceded the crossover, the intermediate haplotype (C22) would be predicted to experience the *same* selective pressure as C11 (Figure S4B). Because C11 is derived by recombination between C3 and C22 in this model, C22 would be expected to predominate over C11, unless C11 had a selective advantage over C22. This outcome is not what is observed. Rather, the frequency of C22 is only approximately 1% that of C11. Furthermore, association with diverse B-region haplotypes rather than one makes it most likely that the existing instances of C22 are the result of recombination after the formation of C11 rather than relicts of a precursor to C11. We conclude that the crossover most likely preceded the *A111T* mutation.

### Phylogeographic analysis of *SLC24A5* haplotype distributions

The world distribution of core region haplotypes, together with their phylogenetic relationships, suggests which haplotypes likely originated in Africa and which most likely arose outside of Africa. As expected from the near fixation of *A111T* in Europe, the C11 clade predominates there, and all other haplotypes are rare. Of the remaining 10 common core haplotype groups, all ancestral at *rs1426654*, eight clearly have their origins in Africa (Figure 3B, Figure 4, and Table S4). Three early diverging haplotypes, C1, C2, and C4, are rare outside of Africa and clearly originated there. In the lineage containing the majority of haplotypes, each of the three branches, containing C5, C6-C7, and C8-C11, give strong evidence of having originated in Africa. C5 reaches its greatest abundance in West Africa and is rare outside of Africa. Within the other two branches, C6 and C9, which are the most common haplotypes in Africa, are also common worldwide, whereas C7 is abundant in East Asia and much less common but widespread in Africa. Consideration of the relationships among haplotype variants (Figure 4) indicates that C6, C7, and C9 (but not C8) dispersed out of Africa and have diverse descendants present and originating in East Asia. Among these descendants is C10, which is abundant in East Asia (and the New World) but extremely rare in Africa (0.5% in LWK). Haplotype C3 represents the final early diverging lineage (Figure 4). Although the lineage containing this haplotype must have originated in Africa, C3 is rare in Africa (1.0% in MKK) but widely distributed in East Asia, the New World, and Oceania. The distributions of C3 and C10 are most consistent with origin outside of Africa and subsequent introduction into Africa by migrations such as those documented by uniparental markers (Richards *et al.* 2006).

### Can we date the *A111T* mutation?

The preceding analysis is consistent with a wide range of possible dates for the origin of *A111T*, including the period before the initial colonization of Europe by anatomically modern humans >40 thousand years ago (kya) (Mellars 2006). An estimate for the date of origin of *A111T* based on microsatellites (Beleza *et al.* 2012) places the origin at 19 kya (95% confidence interval 6−38 kya), for a dominant model, or 11 kya (95% confidence interval 1−56 kya), for a more plausible additive model. To create an independent estimate, we applied a molecular clock approach to 1000 Genomes data by using the combined C and D subregions. Because proportions of different classes of nucleotide substitutions in the C11 + D4 variants and in the human-chimpanzee alignment are not significantly different (χ^2^ = 4.42, df = 5, *P* = 0.49; Table S15), we combined these classes for analysis. For the combined population samples, before making corrections for undercounts in the source data, we obtained an estimate of 7.8 kya for the most recent common ancestor of the C11 + D4 haplotype combination (Table 3). Corresponding 95% confidence limits are 4.8−12.2 kya, whereas uncorrected estimates derived from individual European samples or the combined New World samples (also of European origin) ranged from 5.2 to 10.4 kya (Table 3). These values are clearly underestimates as a result of low sequence depth (1000 Genomes Project Consortium 2012). Adjustment for undercounting is substantial, increasing the estimated age for the combined samples to 12.4 (95% confidence interval 7.6−19.2) kya. If mutation rates in recent humans are lower than predicted from the human-chimpanzee divergence (Scally and Durbin 2012), true ages will be even older. Our adjusted dates overlap those previously reported (Beleza *et al.* 2012) and are also consistent with the lower limit for the origin of *A111T* set by the finding that the Alpine “iceman” dated to 5.3 kya was homozygous for this variant (Keller *et al.* 2012). This date range implies an origin clearly preceding the Neolithic transition in Europe. These dates are later than the initial colonization of Europe but are consistent with an *A111T* origin before or after post-glacial population expansions.

**Table 3:** *A111T* date estimation using 1000 Genomes Project data

Population	Chromosomes	Difference Total	Effective Sample Size	Date, kya (95% Confidence Limits)
Combined	1013	822	19	7.8 (4.8−12.2)
CEU	165	110	13	6.4 (3.4−11.4)
GBR	178	136	14	7.4 (4.1−12.4)
FIN	180	160	10	8.6 (4.5−15.1)
TSI	193	105	18	5.2 (2.9−9.0)
IBS	28	24	9	8.3 (4.2−15.0)
PUR+MXL+CLM	231	249	16*^a^*	10.4 (6.5−15.8)

Human-chimpanzee alignment contains 1227 differences in aligned length of 125,531, of total 127,419 nt in C+D region. When a divergence date of 6 million years ago is used, this corresponds to a per-site mutation rate of 8.1 × 10^−10^ year^-1^. Dates shown are not corrected for undercounting of rare polymorphisms. For combined C11 sample, distribution of variants suggests that tabulated dates should be multiplied by a factor of 1.58. kya, thousand years ago.

a Estimated effective samples sizes ranged from 9-13 for individual admixed New World samples.

Obtaining a better date for the origin of the *A111T* mutation is challenged by a number of issues. Our approach provides a date for the common ancestor of the sampled C11-D4−containing chromosomes. This common ancestor may be significantly younger than the origin of *A111T* if positive selection was initially weak or nonexistent, or if there was a subsequent bottleneck. In addition, our date estimation relies on samples of predominantly European origin. Inclusion of Middle Eastern or South Asian examples would be expected to yield a more representative result. Incomplete detection of rare variants is a limitation that can be improved with higher-coverage sequencing. More direct limits on the age of *A111T* could result from genotyping of ancient human DNA.

### Where did C11 originate?

The precursors to C11, haplotypes C3 and C10, are common in East Asia and the New World (Figure S5), but the distribution of C11 indicates that these locations are not likely sites for the origin of C11 or its immediate precursor. Similarly, B6 not associated with C11 is distributed widely in East Asia and the New World (data not shown). The paucity of C3 and C10 among existing African haplotypes suggests that both events leading to the origin of C11 took place outside this continent. Our dating for this haplotype is consistent with a non-African origin. The most likely location for the origin of C11 is, therefore, within the region in which it is fixed or nearly so. As both models for the origin of C11 imply that C3 and C10 were present in ancestors of Europeans, the observed and inferred distributions of these autosomal haplotypes are consistent with the single-out-of-Africa hypothesis derived using uniparental markers (Oppenheimer 2003; Macaulay *et al.* 2005).

The presence in Africa of *A111T* only in association with C11 indicates that the observed examples, like those of C3 and C10, resulted from introduction into the continent subsequent to origin. The low diversity of B-region haplotypes associated with C11 in MKK, equivalent to that seen in European samples (Figure 5 and File S2) supports this view because those individuals live among a majority population with high B-region diversity. Although too few African C11 sequences have been determined to draw strong conclusions, those available from the 1000 Genomes Project show no evidence of greater age in the form of greater SNP diversity than the European examples. It should be noted that the relatively high abundance of *A111T* in several equatorial East African samples indicates the absence of sustained strong negative selection against this allele at low latitudes.

Although a non-African origin for C11 is clear, near fixation of this haplotype over a wide geographical region prevents strong inferences regarding a precise location of origin. Existing data are consistent with a model in which the C11 precursor did not extend outside the geographical region in which C11 is now nearly fixed, a conclusion subject to limited haplotype sampling in some neighboring regions, such as India. With sufficiently strong positive selection for C11, it is possible that this haplotype could have originated anywhere within its current range and spread via local migration. However, selection acting in concert with major population migrations would have facilitated a much more rapid dispersal. Archeological, mitochondrial, and Y-chromosomal data suggest involvement of multiple dispersals in shaping the current populations of Europe and the Middle East (Soares *et al.* 2010). Because *A111T* is far from fixation in most Indian samples (Table S1), the high diversity of B-region haplotypes associated with C11 in the GIH sample may be the result of prolonged recombination rather than early arrival of *A111T*. In fact, the decrease in frequency of *A111T* to the east of Pakistan suggests that C11 originated farther to the west and after the initial genetic split between western and eastern Eurasians. On this basis, we hold the view that an origin of C11 in the Middle East, broadly defined, is most likely.

Traditionally, uniparental markers have been used for the construction of phylogenetic trees due to the absence of recombination. Here, autosomal haplotypes and the characterization of recombination events have helped us to define the genotypic phylogeny of a genomic region known to have been subject to strong natural selection. Such an approach to studying the phylogeny of autosomal regions may also be useful for the study of other loci under selection in humans and in other organisms.

## Supplementary Material

Supporting Information
