# Supplementary material for: Molecular Phylogeography of a Human Autosomal Skin Color Locus Under Natural Selection
Source: G3 (Bethesda). 2013 Nov 1;3(11):2059–67. doi: 10.1534/g3.113.007484 (PMC3815065; doi:10.1534/g3.113.007484)
Supplement: Supporting Information [file supp_3_11_2059__index.html]

Molecular Phylogeography of a Human Autosomal Skin Color Locus Under Natural Selection — Supporting Information 

# Molecular Phylogeography of a Human Autosomal Skin Color Locus Under Natural Selection

## Supporting Information for Canfield *et al.*, 2013

**Files in this Data Supplement:**

- Supporting Information - Figures S1-S5, Tables S1-S15, and Files S1-S4 (PDF, 1 MB)
- Figure S1 - Common local haplotypes in regions outside core region. (PDF, 342 KB)
- Figure S2 - Relationships between local haplotypes in C and D subregions. (PDF, 405 KB)
- Figure S3 - Association of A-region haplotypes with core haplotype C11. (PDF, 354 KB)
- Figure S4 - Evolutionary history of the C11 haplotype. (PDF, 459 KB)
- Figure S5 - World distributions of haplotype C3 and C10. (PDF, 694 KB)
- Table S1 - *A111T* frequencies used to plot world distribution. (PDF, 454 KB)
- Table S2 - HapMap SNPs used for analysis. (PDF, 325 KB)
- Table S3 - Description of core haplotypes determined using 16 SNPs. (PDF, 363 KB)
- Table S4 - Population distribution of core haplotypes determined using 16 SNPs. (PDF, 348 KB)
- Table S5 - Core haplotypes in HGDP and other samples. (PDF, 337 KB)
- Table S6 - Distribution of core haplotypes in HGDP and other samples. (PDF, 354 KB)
- Table S7 - Apparent fragmentation of core-region haplotypes varies with SNP frequency. (PDF, 309 KB)
- Table S8 - Description of B region haplotypes. (PDF, 316 KB)
- Table S9 - Population distribution of B region haplotypes. (PDF, 322 KB)
- Table S10 - Description of D region haplotypes. (PDF, 324 KB)
- Table S11 - Population distribution of D region haplotypes. (PDF, 329 KB)
- Table S12 - Description of A region haplotypes. (PDF, 330 KB)
- Table S13 - Population distribution of A region haplotypes. (PDF, 330 KB)
- Table S14 - Haploytpe combinations in HGDP and other samples. (PDF, 324 KB)
- Table S15 - Distribution of nucleotide substitutions in C and D regions. (PDF, 307 KB)
- File S1 - Core region haplotypes defined by polymorphisms with MAF greater than or equal to 1% in 1000 Genomes Project data. (.txt, 96 KB)
- File S2 - Haplotype combinations in regions B and C. (.txt, 2 KB)
- File S3 - Haplotype combinations in regions C and D. (.txt, 2 KB)
- File S4 - Haplotype combinations in regions A and C. (.txt, 2 KB)
