# Supplementary material for: Molecular Phylogeography of a Human Autosomal Skin Color Locus Under Natural Selection
Source: G3 (Bethesda). 2013 Nov 1;3(11):2059–67. doi: 10.1534/g3.113.007484 (PMC3815065; doi:10.1534/g3.113.007484)
Supplement: Supporting Information [file supp_g3.113.007484_007484SI.pdf]

G3·Genes | Genomes | Genetics

Molecular phylogeography of a human autosomal skin color locus under natural selection

Victor A. Canfield[*], Arthur Berg[§,†], Steven Peckins[‡,**], Steven M. Wentzel[‡,**],
Khai Chung Ang[‡,**], Stephen Oppenheimer[§§] and Keith C. Cheng[*,§,**]

[*] Department of Pharmacology, Penn State College of Medicine, Hershey, PA 17033
[§] Penn State Cancer Institute, Penn State College of Medicine, Hershey, PA 17033
[†] Department of Public Health Sciences, Penn State College of Medicine, Hershey, PA 17033
[‡] Jake Gittlen Cancer Research Foundation, Penn State College of Medicine, Hershey, PA 17033
[**] Division of Experimental Pathology, Penn State College of Medicine, Hershey, PA 17033
[§§] School of Anthropology and Museum Ethnography, Oxford University, Oxford, OX2 6PE, UK

DOI: 10.1534/g3.113.007484

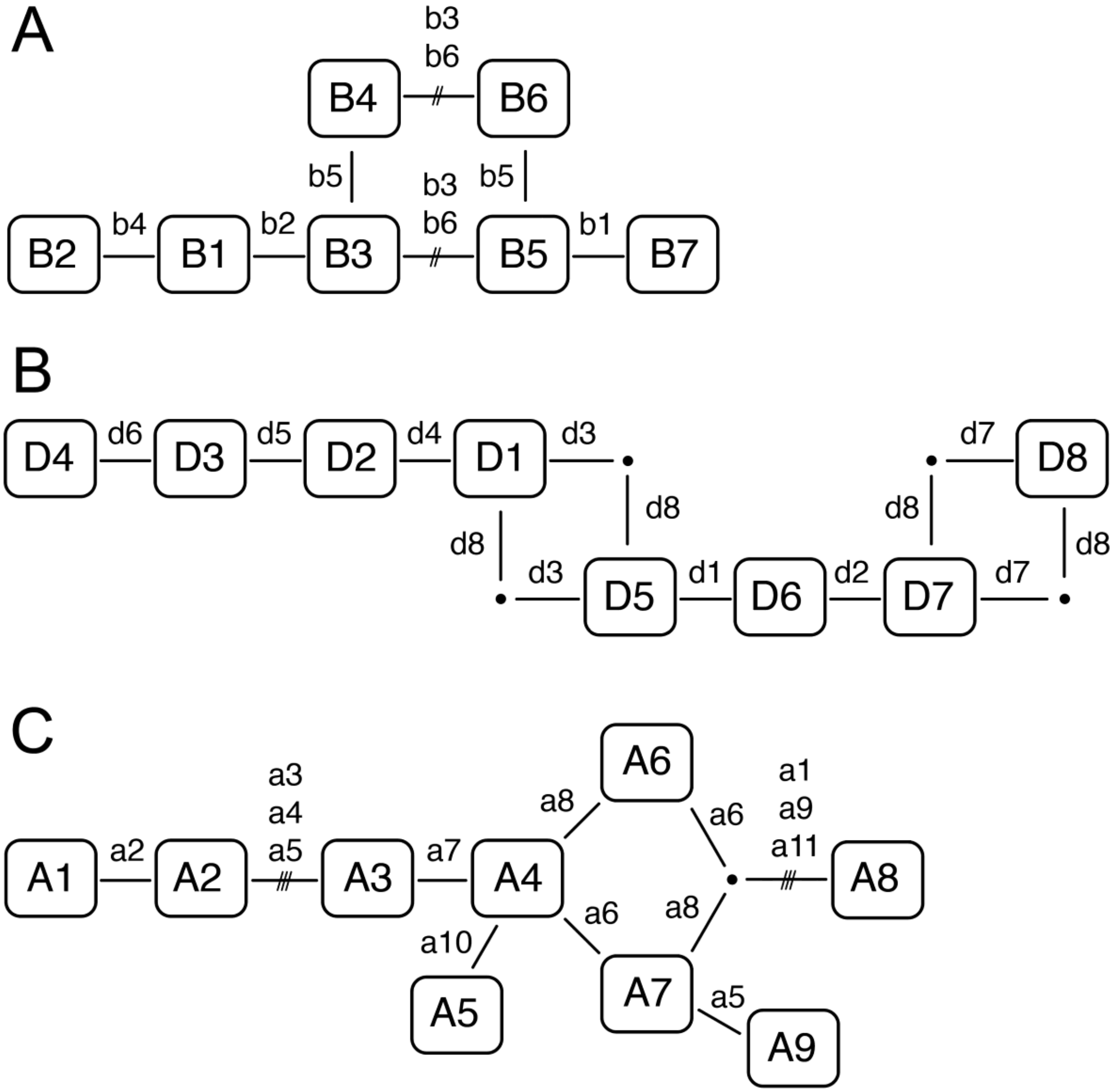

Figure S1 Common local haplotypes in regions outside core region
(A) Diagram showing haplotypes in B-subregion, defined using 6 SNPs.
(B) Haplotypes in D-subregion, defined using 8 SNPs.
(C) Haplotypes in A-subregion, defined using 11 SNPs.
The SNPs contributing to these haplotypes are identified in Table S2. Haplotypes carrying ancestral alleles of indicated SNPs are B1, D1, and A9. Full descriptions of haplotypes are in Tables S8-S13.

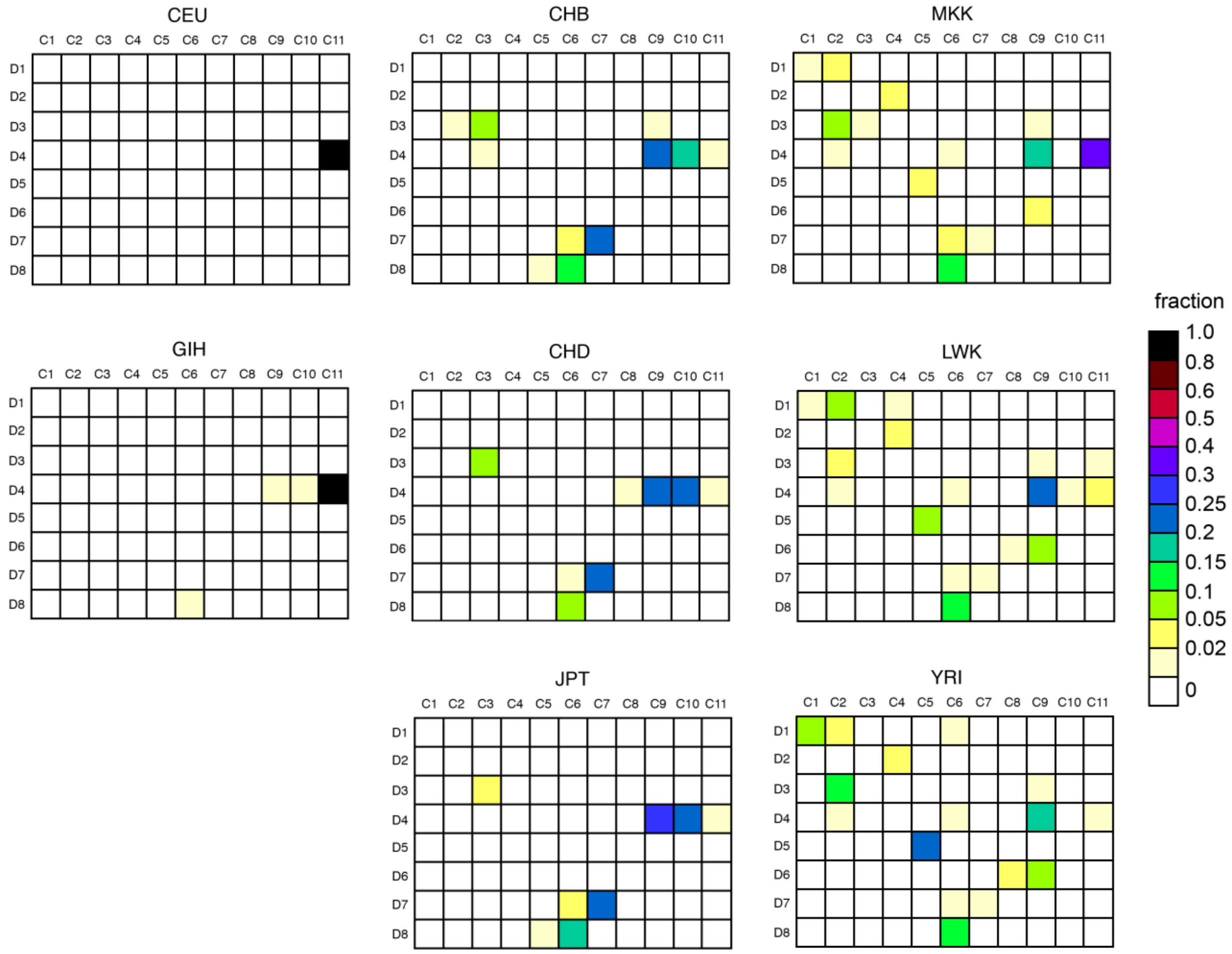

Figure S2 Relationships between local haplotypes in C and D subregions
For each HapMap population, distribution of combinations is shown as heat map (scale at right). Only common haplotypes are shown. Association of C5, C6, C7 and some African instances of C9 with D5-D8, while most members of the C9, C10, and C11 clusters associate with D4, indicates that a crossover between the C- and D-regions occurred in a chromosome that was ancestral to C10 and C11. Recurrent recombination between the core (C) region and D-region is apparent, as is the predominant association of C9, C10, and C11 with D4. Counts are shown in File S2.

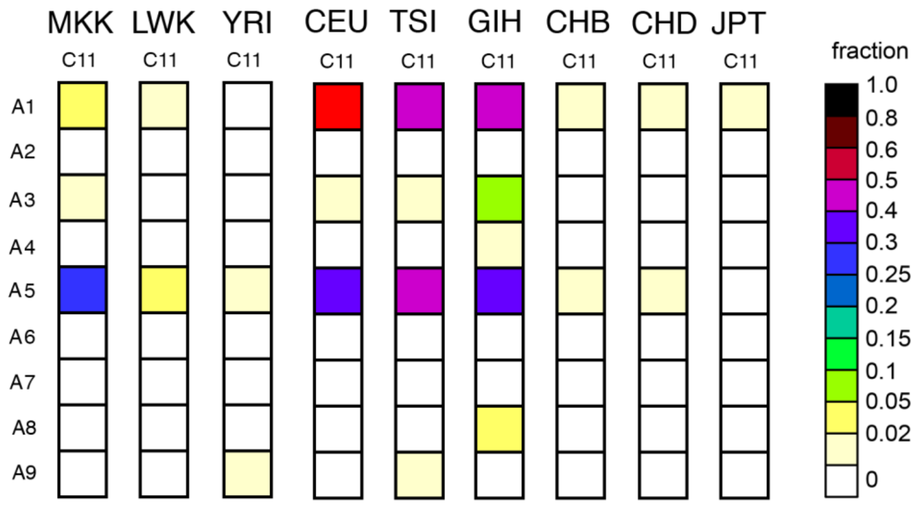

Figure S3 Association of A-region haplotypes with core haplotype C11
Scale (heat map, right) is relative to the total sample in each population, as in Figures 4 and S3. A1 and A5 are most commonly associated with C11. Counts are shown in File S3.

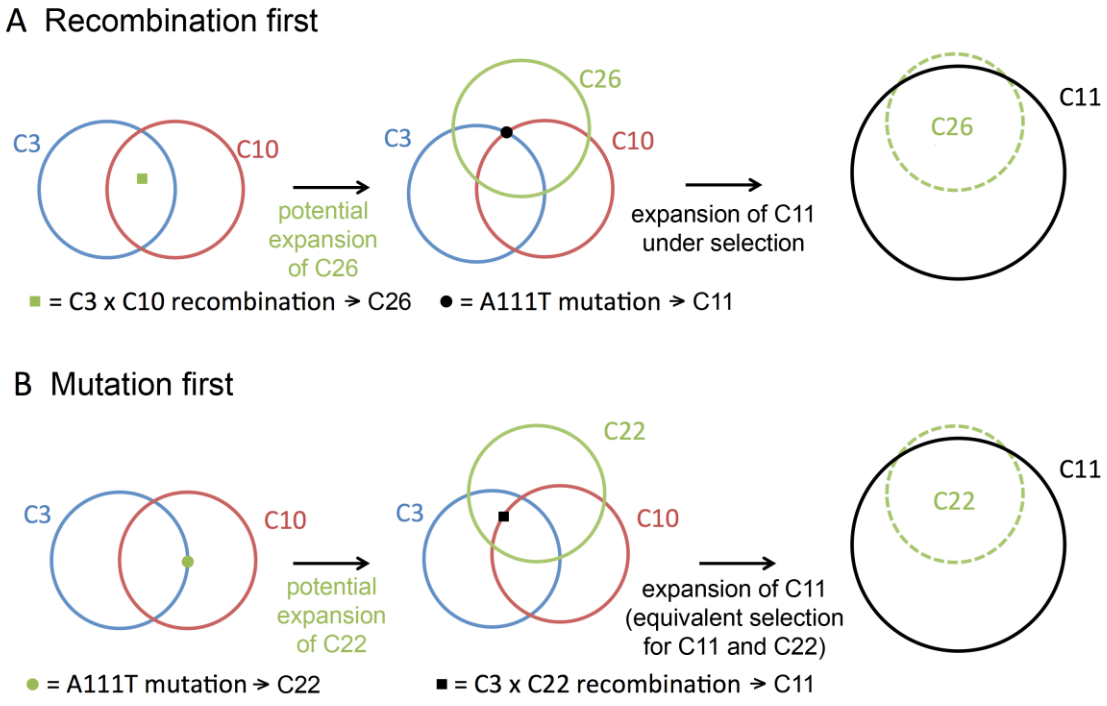

Figure S4 Evolutionary history of the C11 haplotype

Circles represent populations or geographical regions in which various haplotypes are found at successive time intervals. Dots indicate locations of events that created new haplotypes; dots on boundaries represent events that could have occurred in any of the adjoining regions. Diagrams show all potential patterns of co-occurrence, but are not intended to imply that all existed. Geographical regions at different time points may differ as a result of migration, selection, or genetic drift. **A.** Recombination first model. First diagram shows C3 and C10 with overlapping distributions. The green dot indicates recombination between C3 and C10 within their region of overlap to produce haplotype C26, a C11 precursor lacking the *A111T* mutation. At a subsequent time (second diagram), potential expansion of the range of C26 is shown. Creation of C11 by mutation in C26 at *A111T* (black dot) is independent of the distributions of C3 and C10. The third diagram represents the present. C11 expansion as a result of positive selection results in replacement of C26 within the region of fixation. C26 may potentially still exist outside the region in which C11 is fixed. **B.** Mutation first model. First diagram shows C3 and C10. Creation of C22 from C10 by the mutation *A111T* (green dot) is independent of the distribution of C3. At a subsequent time (second diagram), potential expansion or migration of C22 is shown. Recombination between C3 and C22 to produce C11 (black dot) is then independent of C10. The third diagram represents the present. C11 has expanded, while C22 has been reduced to a minor component. Since both haplotypes carry the *A111T* mutation

that is the target of selection, replacement in this model occurs via genetic drift or as a result of a small selective advantage (of unknown origin) of C11 over C22.

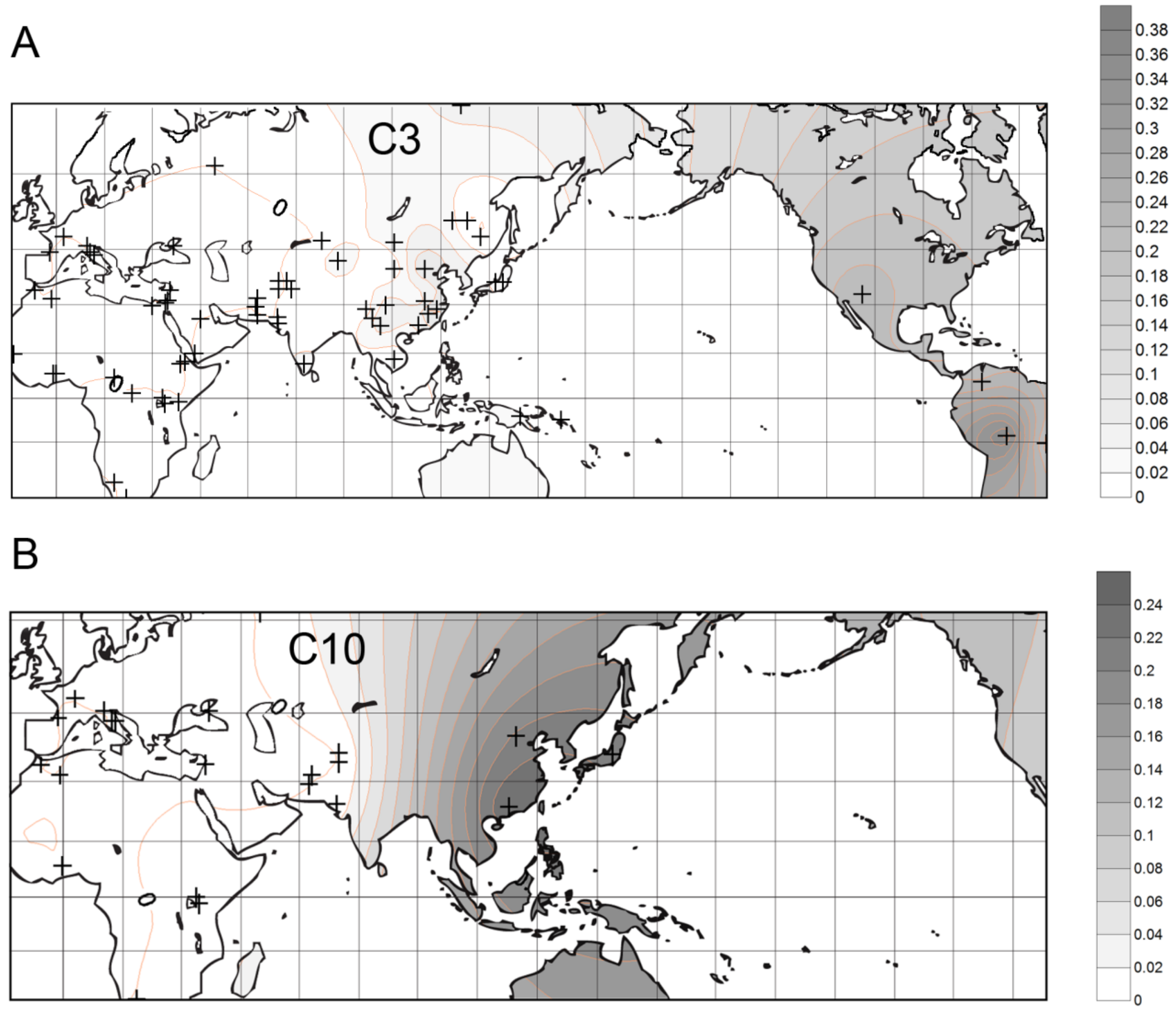

Figure S5 World distributions of haplotype C3 and C10
(A) Contour plot showing frequencies of haplotype C3
(B) Plot of frequency of haplotype C10
Scales are at right of each panel. Sampled populations are indicated (+).

Table S1 ***A111T*** **frequencies used to plot world distribution**

population name or code	sample size (2N)	*A111T* frequency	reference
Hezhen	18	0.000	A
Pima, Mexico	106	0.000	B
Koreans	106	0.000	B
Ticuna	130	0.046	B
Ami	80	0.000	B
Mbuti	78	0.000	B
Han	116	0.017	B
Japanese	98	0.020	B
Melanesian, Nasioi	46	0.000	B
Atayal	84	0.000	B
Karitiana	106	0.000	B
Micronesians	74	0.068	B
Bantu Kenya	24	0.000	A
Tujia	20	0.000	A
Yizu	20	0.000	A
Miaozu	20	0.000	A
Dai	20	0.000	A
Lahu	20	0.000	A
She	20	0.000	A
Naxi	20	0.000	A
Papuan	34	0.000	A
Orcadian	32	1.000	A
Columbian	26	0.000	A
Belorussian	18	0.944	C
Lithuanians	20	1.000	C
Biaka	138	0.007	B
Pima, Arizona	100	0.010	B
Danes	102	0.980	B
Surui	90	0.033	B
Hungarian	178	0.978	B
Japanese, JPT	172	0.012	D
Hakka	80	0.013	B
Yoruba	154	0.013	B
Yoruba, YRI	226	0.013	D
Irish	230	1.000	B
Finns	72	0.931	B
Maya, Yucatan	94	0.128	B
European Americans, CEU	116	1.000	D
Cheyenne	112	0.045	B
Han, CHB	168	0.030	D
Han	98	0.020	B
Lao Loum	238	0.034	B
Northern Italian	28	1.000	A

continued

Table S1 (continued)

population name or code	sample size (2N)	*A111T* frequency	reference
Ibo	96	0.042	B
Komi-Zyrian	92	0.913	B
Russians	66	1.000	B
Yakut	102	0.206	B
Mongola	20	0.150	A
Tu	20	0.050	A
Georgians	40	1.000	C
Europeans, Mixed	182	0.989	B
Russians	96	1.000	B
Bantu Southern	16	0.060	A
French Basque	48	1.000	A
Tuscan	16	1.000	A
Spaniards	115	0.987	B
African Americans	178	0.236	B
San	14	0.070	A
Chuvash	84	0.929	B
Hungarians	40	1.000	C
Cambodians, Khmer	50	0.080	B
Quechua	46	0.152	B
Zaramo	80	0.088	B
French	58	1.000	A
Romanians	32	0.938	C
Daur	20	0.100	A
Hausa	78	0.115	B
Chuvash	34	0.971	C
Sandawe	76	0.118	B
Khanty	98	0.806	B
Orogen	20	0.150	A
Mandenka	48	0.160	E
Adygei	108	0.982	B
Xibo	18	0.190	A
Uzbeks	30	0.600	C
Uygur	20	0.500	A
Spaniards	24	1.000	C
Chagga	90	0.267	B
Masai	40	0.275	B
Tamil	116	0.293	F
Lezgins	36	1.000	C
Armenians	38	1.000	C
Sardinian	56	0.980	A
Adygei	34	1.000	A
Cypriots	24	1.000	C
Turks	38	0.895	C
Syrians	32	1.000	C
Palestinian	102	0.990	A

continued

Table S1 (continued)

population name or code	sample size (2N)	*A111T* frequency	reference
Algerian	68	0.912	B
Kalash	50	1.000	A
Ethiopian Jewish	26	0.460	C
Lebanese	14	1.000	C
South Indian	38	0.470	C
Mozabite	60	0.870	A
Sinhalese	108	0.500	F
Druze	204	1.000	B
Jordanians	40	1.000	C
Hazara	48	0.610	A
Ethiopian	38	0.550	C
Jews, Ethiopian	64	0.578	B
Moroccans	96	0.979	B
Tunisian	194	0.923	B
Moroccan	20	0.900	C
Samaritans	78	1.000	B
Burusho	50	0.980	A
Libya	62	0.919	B
Egypt	24	0.880	C
Yemen	20	0.650	C
Iranians	40	0.925	C
Pathan (Pashtun)	48	0.960	A
Sindhi	50	0.830	A
Keralite	60	0.733	B
Bedouin	96	0.970	A
Brahui	50	1.000	A
Makrani	50	0.920	A
Balochi	50	0.980	A
Saudi	40	0.950	C
Maasai, MKK	286	0.329	D
Luhya, LWK	180	0.067	D
India, AA-C-IP5	36	0.110	G
India, AA-E-IP3	44	0.210	G
India, DR-C-IP2	40	0.020	G
India, DR-S-IP4	42	0.560	G
India, DR-S-LP2	44	0.440	G
India, DR-S-LP3	38	0.390	G
India, IE-E-IP1	44	0.270	G
India, IE-E-LP2	46	0.660	G
India, IE-E-LP4	44	0.610	G
India, IE-N-IP2	46	0.280	G
India, IE-N-LP1	42	0.630	G
India, IE-N-LP5	46	0.710	G
India, IE-N-LP9	46	0.560	G
India, IE-N-SP4	46	0.610	G

continued

Table S1 (concluded)

population name or code	sample size (2N)	*A111T* frequency	reference
India, IE-NE-IP1	44	0.260	G
India, IE-NE-LP1	44	0.480	G
India, IE-W-LP1	42	0.610	G
India, IE-W-LP2	42	0.600	G
India, IE-W-LP3	46	0.590	G
India, IE-W-LP4	44	0.690	G
India, TB-N-IP1	46	0.220	G
India, TB-N-SP1	46	0.360	G
India, TB-NE-LP1	44	0.220	G

References:
A, NORTON et al. 2007
B, CHEUNG et al. 2000, alfred.med.yale.edu/alfred/
C, BEHAR et al. 2010
D, ALTSHULER et al. 2010, http://hapmap.ncbi.nlm.nih.gov/
E, http://www.cephb.fr/en/cephdb/
F, SOEJIMA and KODA 2007
G, INDIAN GENOME VARIATION CONSORTIUM 2008, http://igvdb.res.in

Table S2 HapMap SNPs used for analysis

nickname	SNP_ ID	position (a)	alleles (b)	notes
		**A region**		
**nickname**	**SNP_ ID**	**position (a)**	**alleles (b)**	**notes**
a1	rs16960508	46108528	C/T	(e)
a2	rs1869453	46111620	G/A	(e)
a3	rs1426656	46114468	A/C	
a4	rs17340116	46114858	A/G	
a5	rs1453857	46116200	C/T	(e)
a6	rs2469594	46121836	G/T	
	rs2470104	46122159	T/C	(e)
a7	rs1025199	46126798	C/A	
a8	rs1365453	46129898	A/C	
a9	rs991877	46137615	T/C	(e)
a10	rs2433363	46139544	G/A	(e)
a11	rs16960535	46147951	T/C	
		**B region**		
**nickname**	**SNP_ ID**	**position**	**alleles**	**notes**
b1	rs16960541	46157395	G/T	
b2	rs2433360	46161326	A/C	
b3	rs2459392	46163796	G/A	
	rs10519163	46164805	C/T	(e)
b4	rs7180657	46168230	T/C	(e)
	rs2459383	46171162	G/A	(e)
b5	rs2250072	46172199	G/A	(e)
b6	rs2459385	46174380	C/A	(e)
		**C (core) region**		
**nickname**	**SNP_ ID**	**position**	**alleles**	**notes**
	rs12440301	46177216	G/A	(e)
	rs12441154	46178248	C/T	(f)
c1	rs1834640	46179457	G/A	(c, e)
	rs1559857	46184100	A/G	(e)
c2	rs2675345	46187491	G/A	(f)
c3	rs2469592	46189167	G/A	(e)
c4	rs2470101	46191159	C/T	(e)
c5	rs938505	46193187	C/T	(e)
c6	rs2433354	46202261	T/C	(e)
c7	rs2459391	46202360	G/A	(f)
c8	rs2433356	46203652	A/G	(e)
	rs16960620	46204191	A/G	(f, h)
c9	rs2675347	46205937	G/A	(e, g)
	rs2555364	46206678	C/G	(f, h)

continued

Table 2 (concluded)

c10	rs2675348	46208036	G/A	(e)
	rs8040016	46212039	C/T	(e)
	rs16960624	46212505	G/A	(e)
c11	rs1426654	46213776	G/A	(f, h)
c12	rs2470102	46220786	G/A	(f)
c13	rs16960631	46226209	A/G	(c, d, f)
c14	rs2675349	46232679	G/A	(f)
	rs3736482	46237809	T/C	(e)
	rs9652449	46240091	T/G	(e)
c15	rs3817315	46248438	T/C	(f)
c16	rs7163587	46255311	T/C	(f)
		**D region**		
**nickname**	**SNP_ ID**	**position**	**alleles**	**notes**
d1	rs2413886	46265512	T/G	(c, e)
d2	rs34722053	46268957	C/T	(c)
d3	rs8037482	46275588	A/G	(c, e)
d4	rs7170260	46288372	G/T	(c)
d5	rs1878186	46295692	C/T	(c, e)
d6	rs9920281	46301601	G/A	(e)
d7	rs8032941	46302144	T/C	(c)
d8	rs16960679	46304763	G/A	(c, d)

SNPs given nicknames (named SNPs) were genotyped and phased in all 11 HapMap phase 3 populations (release 27), except as otherwise noted. SNPs genotyped in HapMap phase 2 (release 21) are omitted here unless cited elsewhere.

Footnotes:
(a) NCBI B36 coordinates; for B37, add 2212708
(b) ancestral allele first
(c) named SNPs absent from TSI
(d) named SNPs absent from MEX
(e) present in HGDP
(f) core-region SNP absent from HGDP data
(g) absent from HapMap phase 2 release 21
(h) genotyped in GIARDINA *et al.* (2008)

Table S3 Description of core haplotypes determined using 16 SNPs

haplotype		SNP															
number (a)	name	c1	c2	c3	c4	c5	c6	c7	c8	c9	c10	c11	c12	c13	c14	c15	c16
18	C1	G	G	G	C	C	T	G	A	G	G	G	G	A	G	T	T
16	C2	G	G	G	C	T	T	G	A	G	G	G	G	A	G	T	T
17	C3	A	G	G	C	T	T	G	A	G	G	G	G	A	G	T	T
20	C4	G	G	G	T	C	T	G	A	G	G	G	G	A	G	T	T
19	C5	G	G	A	T	C	T	A	A	G	G	G	G	A	G	T	C
12	C6	G	G	A	T	C	T	A	G	G	G	G	G	A	G	T	C
22	C7	G	G	A	T	C	T	A	G	G	G	G	G	G	G	T	C
28	C8	G	G	A	T	C	C	A	G	A	G	G	G	A	A	C	C
8	C9	G	G	A	T	C	C	A	G	A	A	G	G	A	A	C	C
13	C10	G	A	A	T	C	C	A	G	A	A	G	A	A	A	C	C
1	C11	A	A	A	T	C	C	A	G	A	A	A	A	A	A	C	C
2	C25	A	A	A	T	T	C	A	A	A	A	A	A	A	A	C	C
3	C24	A	A	A	T	C	C	A	A	A	A	A	A	A	A	C	C
4	C23	A	A	A	T	C	C	A	G	A	A	A	A	A	A	T	C
5	(b)	A	A	A	T	T	C	G	A	G	A	A	G	A	G	C	C
7		A	G	A	T	C	C	A	G	A	A	A	A	A	A	C	C
9		G	G	A	T	C	C	A	G	A	A	A	A	A	A	C	C
10	(b)	A	A	A	T	C	C	A	G	A	A	A	A	A	G	T	C
15	(b)	A	G	G	C	T	C	G	A	A	A	A	A	A	A	C	C
25	C22	G	A	A	T	C	C	A	G	A	A	A	A	A	A	C	C
37		G	G	A	T	C	T	A	A	G	G	A	G	A	G	T	C
41		A	G	G	C	T	T	G	A	G	G	A	G	A	G	T	T
46		G	A	A	T	C	C	A	G	A	A	A	A	A	A	T	C
6	(b)	A	G	G	C	C	T	A	G	A	G	G	A	A	A	T	T
11	(b)	G	G	A	T	C	T	A	G	G	G	G	G	G	A	C	C
14	(b)	G	A	A	T	C	T	A	G	G	G	G	G	A	G	T	T
21	C20	G	G	A	T	C	C	A	G	A	A	G	G	A	G	T	C
23	C12	G	G	G	C	T	T	G	A	G	G	G	G	A	G	T	C
24		G	G	G	C	C	T	G	A	A	G	G	G	A	G	T	T
26	C17	G	G	A	T	C	T	G	G	G	G	G	G	A	G	T	C
27	C15	G	G	A	T	C	T	G	A	G	G	G	G	A	G	T	T
29	C16	G	G	A	T	C	T	G	A	G	G	G	G	A	G	T	C
30	C13	G	G	G	C	T	T	G	G	G	G	G	G	A	G	T	T
31		G	G	A	T	T	C	A	G	A	G	G	G	A	A	C	C
32		G	G	G	C	C	T	G	G	G	G	G	G	A	G	T	C
33	C14	G	G	G	C	T	T	G	G	G	G	G	G	A	G	T	C
34		G	G	G	T	C	C	A	G	A	A	G	G	A	A	C	C
35		G	G	A	T	C	T	A	G	A	G	G	G	A	G	T	C
36		G	G	A	T	C	T	A	G	A	G	G	G	G	G	T	C
38		G	G	A	T	C	C	A	G	A	A	G	G	G	G	C	C
39	C18	G	G	A	T	C	C	A	G	A	A	G	G	A	A	T	C

continued

Table S3 (concluded)

haplotype		SNP															
number (a)	name	c1	c2	c3	c4	c5	c6	c7	c7	c9	c10	c11	c12	c13	c14	c15	c16
40		G	G	A	T	C	T	A	A	G	A	G	G	A	G	T	C
42	C21	G	G	A	T	C	C	A	G	A	A	G	A	A	A	C	C
43		G	G	A	T	C	C	A	G	A	G	G	G	A	G	C	T
44		A	G	A	T	C	T	A	G	G	G	G	G	A	G	T	C
45		G	G	A	T	C	T	A	G	G	G	G	G	A	A	C	C
47		G	A	A	T	C	C	A	G	A	A	G	G	G	A	T	C
48		A	G	G	C	T	T	G	A	A	G	G	G	A	G	T	T
49		G	A	A	T	C	C	A	A	A	A	G	A	A	A	C	C
50	C26	A	A	A	T	C	C	A	G	A	A	G	A	A	A	C	C
51	C19	G	G	A	T	C	C	A	G	A	A	G	G	A	G	C	C
52		G	G	A	T	C	T	A	G	G	G	G	G	A	A	T	C

Footnotes:

(a) Haplotype numbers used only in Tables S3 and S4.

(b) Three misphased pairs are, after correction:

5+6: C11+C2/C3

10+11: C11+C7

14+15: C11+C2

Table S4 Population distribution of core haplotypes determined using 16 SNPs

haplotype			population										
number (a)	name	total	CEU	TSI	GIH	MKK	YRI	LWK	CHB	CHD	JPT	MEX	ASW
18	C1	25	0	0	0	7	13	4	0	0	0	0	1
16	C2	125	0	0	0	38	38	26	1	0	0	1	21
17	C3	45	0	0	0	3	0	0	14	14	5	9	0
20	C4	30	0	0	0	6	7	14	0	0	0	0	3
19	C5	98	0	0	0	13	50	14	1	0	1	0	19
12	C6	245	0	0	1	52	42	33	34	21	35	7	20
22	C7	122	0	0	0	1	3	2	40	39	36	0	1
28	C8	10	0	0	0	0	5	1	0	1	0	0	3
8	C9	365	0	0	3	65	61	63	36	44	51	15	27
13	C10	123	0	0	1	0	0	1	34	41	37	9	0
1	C11	638	109	172	164	92	3	9	3	3	1	60	22
2	C25	2	2	0	0	0	0	0	0	0	0	0	0
3	C24	3	2	0	0	0	0	1	0	0	0	0	0
4	C23	5	2	1	0	0	0	0	1	0	0	1	0
5		1	0	1	0	0	0	0	0	0	0	0	0
7		2	0	1	0	0	0	0	0	0	0	1	0
9		1	0	0	1	0	0	0	0	0	0	0	0
10		2	0	0	2	0	0	0	0	0	0	0	0
15		1	0	0	1	0	0	0	0	0	0	0	0
25	C22	4	0	0	0	2	0	1	1	0	0	0	0
37		1	0	0	0	0	0	1	0	0	0	0	0
41		1	0	0	0	0	0	0	0	1	0	0	0
46		1	0	0	0	0	0	0	0	0	1	0	0
6		1	0	1	0	0	0	0	0	0	0	0	0
11		2	0	0	2	0	0	0	0	0	0	0	0
14		1	0	0	1	0	0	0	0	0	0	0	0
21	C20	5	0	0	0	4	1	0	0	0	0	0	0
23	C12	2	0	0	0	2	0	0	0	0	0	0	0
24		1	0	0	0	1	0	0	0	0	0	0	0
26	C17	8	0	0	0	0	1	5	0	0	0	0	2
27	C15	4	0	0	0	0	2	0	0	0	0	0	2
29	C16	1	0	0	0	0	1	0	0	0	0	0	0
30	C13	1	0	0	0	0	1	0	0	0	0	0	0
31		1	0	0	0	0	1	0	0	0	0	0	0
32		1	0	0	0	0	1	0	0	0	0	0	0
33	C14	3	0	0	0	0	0	2	0	0	0	0	1
34		1	0	0	0	0	0	1	0	0	0	0	0
35		1	0	0	0	0	0	1	0	0	0	0	0
36		1	0	0	0	0	0	1	0	0	0	0	0
38		2	0	0	0	0	0	0	1	1	0	0	0
39	C18	2	0	0	0	0	0	0	1	0	1	0	0

continued

Table S4 (concluded)

haplotype		total	population										
number (a)	name		CEU	TSI	GIH	MKK	YRI	LWK	CHB	CHD	JPT	MEX	ASW
40		2	0	0	0	0	0	0	1	1	0	0	0
42	C21	2	0	0	0	0	0	0	0	2	0	0	0
43		1	0	0	0	0	0	0	0	1	0	0	0
44		1	0	0	0	0	0	0	0	1	0	0	0
45		1	0	0	0	0	0	0	0	0	1	0	0
47		1	0	0	0	0	0	0	0	0	1	0	0
48		1	0	0	0	0	0	0	0	0	1	0	0
49		1	0	0	0	0	0	0	0	0	1	0	0
50	C26	1	0	0	0	0	0	0	0	0	0	1	0
51	C19	3	0	0	0	0	0	0	0	0	0	0	3
52		1	0	0	0	0	0	0	0	0	0	0	1
**total**		**1903**	**115**	**176**	**176**	**286**	**230**	**180**	**168**	**170**	**172**	**104**	**126**

Footnotes:
(a) Haplotype numbers used only in Tables S3 and S4.

Table S5 Core haplotypes in HGDP and other samples

haplotype		SNP (a)															
number (b)	name (c)	c1	'857	c3	c4	c5	c6	c8	c9	c10	'016	'624	c11	'482	'449	HGDP count	other (d)
14	C1	G	A	G	C	C	T	A	G	G	C	G	G	T	T	9	
13	C1	G	A	G	C	C	T	A	G	G	C	G	G	T	G	2	
12	C1	G	A	G	C	C	T	A	G	G	C	G	G	C	G	1	
16	C2	G	A	G	C	T	T	A	G	G	C	G	G	T	G	28	7
18	C2	G	A	G	C	T	T	A	G	G	T	G	G	T	T	6	6
17	C2	G	A	G	C	T	T	A	G	G	C	G	G	T	T	3	1
15	C2	G	A	G	C	T	T	A	G	G	C	G	G	C	G	1	
24	C2	G	A	G	C	T	T	A	G	G	T	G	G	C	G		1
23	C3	A	A	G	C	T	T	A	G	G	T	G	G	T	T	80	1
11	C4	G	A	G	T	C	T	A	G	G	C	G	G	T	T	15	
10	C4	G	A	G	T	C	T	A	G	G	C	G	G	T	G	3	
7	C5	G	A	A	T	C	T	A	G	G	C	G	G	C	G	20	1
8	C5	G	A	A	T	C	T	A	G	G	C	A	G	C	G	19	1
6	C6/ C7	G	A	A	T	C	T	G	G	G	C	G	G	C	G	279	20
2	C8	G	G	A	T	C	C	G	A	G	C	G	G	C	G	4	
1a	C9/ C10	G	G	A	T	C	C	G	A	A	C	G	G	C	G	374	28
5	C9/ C10	G	A	A	T	C	C	G	A	A	C	G	G	C	G	5	
19a	C26	A	G	A	T	C	C	G	A	A	C	G	G	C	G	2	
19d	C11	A	G	A	T	C	C	G	A	A	C	G	A	C	G	1005	133
20	C11	A	A	A	T	C	C	G	A	A	C	G	A	C	G	4	2
1d	C22	G	G	A	T	C	C	G	A	A	C	G	A	C	G	14	
4		G	G	A	T	C	C	G	G	G	C	G	G	C	G	2	
3		G	G	A	T	C	C	G	G	A	C	G	G	C	G	1	
9		G	A	G	T	C	T	G	G	G	C	G	G	C	G	1	2
21		A	A	A	T	C	T	G	G	G	C	G	G	C	G	1	
22		A	A	G	C	T	C	G	A	A	C	G	G	C	G	1	
25		G	A	A	T	C	C	G	A	A	C	G	A	C	G		1
**total**																**1880**	**204**

Footnotes:

(a) SNPs shared with HapMap phase 3 data are identified by nickname; others by last three digits of rs# (Table S2)
(b) haplotype designations specific to this table
(c) equivalents classified using 11 SNPs
(d) data from BEHAR et al. 2010

Table S6 Distribution of core haplotypes in HGDP and other samples

HGDP code	population name	haplotype												total
		C1	C2	C3	C4	C5	C6/ C7	C8	C9/ C10	C26	C11	C22	others	
20	Orcadian										30			30
21	Adygei										34			34
22	Russian										47	3		50
24	Basque										48			48
25	French										55	1		56
27	Italian										24			24
28	Sardinian				1						55			56
29	Tuscan										16			16
34	Mozabite		2			3	2	1			50			58
36	Bedouin		2				1				88	1		92
37	Druze										84			84
38	Palestinian						1		1		90			92
50	Balochi		1								45	2		48
51	Brahui										49	1		50
52	Burusho								1		49			50
54	Hazara		1				6		8		29			44
56	Kalash										46			46
57	Makrani		1				1		2		43	3		50
58	Pathan (Pashtun)						2				41	1		44
59	Sindhi						5		3		39	1		48
71	Melanesian						16		4					20
75	Papuan			2			25		7					34
81	Colombian			5			1		8					14
82	Karitiana			15					13					28
83	Surui			5					11					16
86	Maya			12			6		19		4		1	42
87	Pima			10					18					28
430	Bantu SouthAfrica		4			4	2		5		1			16
441	BantuKenya	1	3		2	3	7		6					22
464	Mandenka	3	2		1	14	12		7		5			44
465	Yoruba	1	3		1	11	9	2	14		1			42
488	BiakaPygmy	3	5		6	2	5		21					42
489	MbutiPygmy	3	5		7	2	1		7				1	26
494	San	1	8								1			10

continued

Table S6 (concluded)

HGDP code	population name	haplotype C1	C2	C3	C4	C5	C6/ C7	C8	C9/ C10	C26	C11	C22	others	total
601	Han			5			22		39	1	1			68
602	Han-NChina			3			7		10					20
606	Dai		1	2			9		8					20
607	Daur			1			7		8		2			18
608	Hezhen						7		8				1	16
611	Lahu			1			8		7					16
612	Miao			3			4		12				1	20
613	Oroqen						6		9		3			18
615	She			3			7		9				1	20
616	Tujia			1			10		9					20
617	Tu						7		12		1			20
618	Xibo						5		9	1	3			18
619	Yi			2			5		13					20
622	Mongola			1			11		5		3			20
625	Naxi						8		8					16
629	Uygur			1			7		2		9	1		20
677	Cambodian						10		8		1		1	20
684	Japanese			4			21	1	30					56
699	Yakut			4			16		18		12			50
	Moroccan					1	1				18			20
	Egyptian		1						2		21			24
	Ethiopian (mixed)		6				4		7		21			38
	Ethiopian Jewish		5				1		6		12		2	26
	Saudi						1		1		37		1	40
	Yemeni		1			1	3		2		13			20
	South Indian (mixed)		2	1			10		10		15			38
**subtypes**		**3**	**5**	**1**	**2**	**2**	**1**	**1**	**2**	**1**	**2**	**1**	**6**	
**total**		**12**	**53**	**81**	**18**	**41**	**299**	**4**	**407**	**2**	**1146**	**14**	**9**	**2086**
**most common subtype**		**9**	**35**	**81**	**15**	**21**	**299**	**4**	**402**	**2**	**1138**	**14**	**4**	

Table S7   Apparent fragmentation of core-region haplotypes varies with SNP frequency.

polymorphism cutoff frequency	total polymorphisms	total haplotypes	common haplotypes	fraction in common haplotypes
0.1	24	104	15	0.92
0.05	73	187	21	0.85
0.02	109	215	21	0.83
0.01	156	291	34	0.78
0.005	239	349	39	0.72
0.0025	287	403	34	0.64
0.0009	504	546	28	0.54
all SNPs	744	653	26	0.52
all (0.045%)	767	716	25	0.48

Common haplotypes are defined as those with frequencies > 0.5% in combined sample. Values here are not corrected for undercounting of rare SNPs.

Table S8 Description of B region haplotypes

haplotype			SNP (a)					
**number (b)**	**name**		**b1**	**b2**	**b3**	**b4**	**b5**	**b6**
		**ancestral state**	**G**	**A**	**G**	**T**	**G**	**C**
1	B6		G	C	A	T	A	A
2	B7		T	C	A	T	G	A
3			G	C	G	C	G	C
4	B2		G	A	G	C	G	C
5	B5		G	C	A	T	G	A
6	B3		G	C	G	T	G	C
7			T	C	A	T	G	C
8	B1		G	A	G	T	G	C
9			T	A	G	T	G	C
10	B4		G	C	G	T	A	C
11			G	A	A	T	A	A
12			T	C	A	T	A	A
13			T	C	G	T	G	A
14			G	C	A	T	G	C
15			G	A	A	T	G	A
16			G	C	A	T	A	C
17			T	A	G	C	G	C
18			T	C	G	T	G	C
19			G	C	G	T	G	A
20			G	C	A	C	G	C
21			G	A	G	C	G	A

Footnotes:
(a) SNPs identified by nickname (Table S2)
(b) Haplotype numbers used only in Tables S8 and S9

Table S9 Population distribution of B region haplotypes

haplotype			population										
**number (a)**	**name**	**total**	**CEU**	**TSI**	**GIH**	**MKK**	**YRI**	**LWK**	**CHB**	**CHD**	**JPT**	**MEX**	**ASW**
1	B6	673	109	167	151	100	2	9	23	20	12	65	15
2	B7	238	4	6	2	65	33	20	21	27	28	14	18
3		4	2	1	0	0	0	0	0	0	0	0	1
4	B2	511	0	2	13	59	106	76	59	61	68	18	49
5	B5	238	0	0	4	37	64	49	18	20	17	3	26
6	B3	137	0	0	5	0	3	0	43	38	42	3	3
7		1	0	0	1	0	0	0	0	0	0	0	0
8	B1	51	0	0	0	14	12	13	1	0	0	0	11
9		5	0	0	0	5	0	0	0	0	0	0	0
10	B4	20	0	0	0	3	10	5	0	0	0	0	2
11		2	0	0	0	2	0	0	0	0	0	0	0
12		3	0	0	0	1	0	0	0	1	0	1	0
13		3	0	0	0	0	0	3	0	0	0	0	0
14		2	0	0	0	0	0	1	0	1	0	0	0
15		2	0	0	0	0	0	2	0	0	0	0	0
16		1	0	0	0	0	0	1	0	0	0	0	0
17		3	0	0	0	0	0	1	1	0	1	0	0
18		2	0	0	0	0	0	0	1	0	1	0	0
19		5	0	0	0	0	0	0	1	1	3	0	0
20		1	0	0	0	0	0	0	0	1	0	0	0
21		1	0	0	0	0	0	0	0	0	0	0	1
**total**		**1903**	**115**	**176**	**176**	**286**	**230**	**180**	**168**	**170**	**172**	**104**	**126**

Footnotes:
(a) haplotype numbers used only in Tables S8 and S9

Table S10 Description of D region haplotypes

haplotype			SNP (a)							
number (b)	name		d1	d2	d3	d4	d5	d6	d7	d8
		ancestral state	T	C	A	G	C	G	T	G
1	D4		T	C	A	T	T	A	T	G
2	D3		T	C	A	T	T	G	T	G
12	D2		T	C	A	T	C	G	T	G
5	D1		T	C	A	G	C	G	T	G
11	D5		T	C	G	G	C	G	T	A
10	D6		G	C	G	G	C	G	T	A
3	D7		G	T	G	G	C	G	T	A
4	D8		G	T	G	G	C	G	C	G
9			G	T	G	G	C	G	T	G
16			G	T	G	G	C	A	T	G
14			G	C	G	G	C	G	T	G
19			T	C	A	T	C	G	T	A
8			T	C	A	G	C	G	T	A
15			G	C	G	G	C	G	C	G
22			T	C	A	T	T	G	C	G
18			T	C	G	G	C	G	T	G
6			T	C	A	G	C	G	C	G
21			T	C	A	T	T	G	T	A
23			T	C	A	T	T	A	T	A
26			T	C	A	G	T	A	T	G
17			T	T	G	G	C	G	T	G
13			T	C	A	T	C	G	C	G
7			T	C	A	G	C	A	T	G
24			G	T	G	G	T	G	T	A
20			T	C	G	G	C	A	T	G
25			T	C	A	T	T	A	C	G
27			G	T	A	T	T	G	T	G
28			G	C	G	G	T	A	T	G
29			G	T	A	T	T	A	T	G
30			G	T	G	G	C	G	C	A
31			G	C	A	T	T	G	C	G
32			T	T	G	G	C	G	C	G
33			T	C	G	G	C	G	C	G
**total**										

Footnotes:
(a) SNPs identified by nickname (Table S2)
(b) Haplotype numbers used only in Tables S10 and S11

Table S11 Population distribution of D region haplotypes

haplotype			population										
**number (a)**	**name**	**total**	**CEU**	**TSI**	**GIH**	**MKK**	**YRI**	**LWK**	**CHB**	**CHD**	**JPT**	**MEX**	**ASW**
1	D4	1080	115	173	172	139	47	52	73	90	92	86	41
2	D3	122	0	3	0	23	27	10	15	15	6	11	12
12	D2	25	0	0	0	6	8	9	0	0	0	0	2
5	D1	67	0	0	1	15	20	17	0	0	0	0	14
11	D5	89	0	0	0	7	50	15	0	0	0	0	17
10	D6	59	0	0	0	14	18	19	0	0	0	0	8
3	D7	154	0	0	2	9	5	5	49	41	41	0	2
4	D8	192	0	0	1	32	34	30	23	17	30	7	18
9		13	0	0	0	10	1	0	1	1	0	0	0
16		13	0	0	0	3	3	4	2	1	0	0	0
14		10	0	0	0	3	2	3	0	0	1	0	1
19		9	0	0	0	1	3	5	0	0	0	0	0
8		8	0	0	0	4	1	3	0	0	0	0	0
15		7	0	0	0	2	2	2	0	0	0	0	1
22		7	0	0	0	0	5	0	0	1	0	0	1
18		7	0	0	0	5	1	0	0	0	0	0	1
6		7	0	0	0	5	1	1	0	0	0	0	0
21		5	0	0	0	0	1	2	0	0	0	0	2
23		5	0	0	0	0	1	2	1	0	0	0	1
26		4	0	0	0	0	0	0	1	2	1	0	0
17		4	0	0	0	4	0	0	0	0	0	0	0
13		3	0	0	0	1	0	0	1	0	0	0	1
7		2	0	0	0	2	0	0	0	0	0	0	0
24		2	0	0	0	0	0	1	0	0	0	0	1
20		1	0	0	0	1	0	0	0	0	0	0	0
25		1	0	0	0	0	0	0	1	0	0	0	0
27		1	0	0	0	0	0	0	1	0	0	0	0
28		1	0	0	0	0	0	0	0	1	0	0	0
29		1	0	0	0	0	0	0	0	1	0	0	0
30		1	0	0	0	0	0	0	0	0	1	0	0
31		1	0	0	0	0	0	0	0	0	0	0	1
32		1	0	0	0	0	0	0	0	0	0	0	1
33		1	0	0	0	0	0	0	0	0	0	0	1
**total**		**1903**	**115**	**176**	**176**	**286**	**230**	**180**	**168**	**170**	**172**	**104**	**126**

Footnotes:
(a) haplotype numbers used only in Tables S10 and S11

Table S12 Description of A region haplotypes

haplotype			SNP (a)										
number (b)	name		a1	a2	a3	a4	a5	a6	a7	a8	a9	a10	a11
		ancestral state	**C**	**G**	**A**	**A**	**C**	**G**	**C**	**A**	**T**	**G**	**T**
1	A1		C	A	C	G	C	T	A	A	T	G	T
2	A5		C	G	A	A	T	T	C	A	T	A	T
3	A3		C	G	A	A	T	T	A	A	T	G	T
4			C	A	C	G	T	T	A	A	T	G	T
5			T	G	A	A	T	G	C	C	T	G	T
6			C	G	A	A	T	T	C	A	C	A	C
7	A9		C	G	A	A	C	G	C	A	T	G	T
8	A8		T	G	A	A	T	G	C	C	C	G	C
9			T	G	A	A	T	T	A	A	T	G	T
10	A4		C	G	A	A	T	T	C	A	T	G	T
11			C	G	A	A	C	G	C	A	T	A	T
12			T	G	A	A	T	G	A	A	T	G	T
13			C	A	C	G	C	T	C	A	T	A	T
14			C	G	A	A	T	G	C	A	T	A	T
15	A6		C	G	A	A	T	T	C	C	T	G	T
16	A7		C	G	A	A	T	G	C	A	T	G	T
17			C	G	C	G	C	T	C	A	T	G	T
18			C	G	A	A	T	T	C	C	C	G	C
19			C	G	C	G	C	T	C	A	T	A	T
20	A2		C	G	C	G	C	T	A	A	T	G	T
21			C	A	A	A	C	G	C	A	T	G	T
22			C	A	C	G	C	T	A	A	T	A	T
23			C	G	A	A	T	T	A	A	T	A	T
24			C	G	A	G	C	G	C	A	T	G	T
25			C	A	A	A	T	G	C	A	T	G	T
26			C	A	A	A	T	T	A	A	T	G	T
27			C	G	A	G	C	T	C	A	T	G	T
28			C	A	C	G	C	T	A	A	T	G	C
29			C	A	C	A	C	T	A	A	T	G	T
30			C	A	A	A	T	T	A	A	T	A	T
31			T	A	A	A	T	G	C	C	C	G	C
32			T	G	A	A	T	G	C	A	T	G	C
33			C	G	A	A	T	T	C	C	C	G	T
34			C	G	A	A	T	T	C	C	T	A	T
**total**													

Footnotes:
(a) SNPs identified by nickname (Table S2)
(b) Haplotype numbers used only in Tables S12 and S13

Table S13 Population distribution of A region haplotypes

haplotype			population										
number (a)	name	total	CEU	TSI	GIH	MKK	YRI	LWK	CHB	CHD	JPT	MEX	ASW
1	A1	660	73	85	93	40	41	22	74	77	79	39	37
2	A5	597	41	83	64	158	57	61	28	26	11	38	30
3	A3	116	1	3	10	3	0	1	33	26	37	2	0
4		3	0	2	0	0	0	0	0	0	0	1	0
5		1	0	1	0	0	0	0	0	0	0	0	0
6		1	0	1	0	0	0	0	0	0	0	0	0
7	A9	225	0	1	0	35	86	61	0	0	0	1	41
8	A8	128	0	0	5	2	2	4	29	36	37	13	0
9		1	0	0	1	0	0	0	0	0	0	0	0
10	A4	25	0	0	1	10	4	7	2	0	0	0	1
11		6	0	0	1	1	2	2	0	0	0	0	0
12		2	0	0	1	0	0	0	0	1	0	0	0
13		17	0	0	0	16	0	1	0	0	0	0	0
14		2	0	0	0	2	0	0	0	0	0	0	0
15	A6	23	0	0	0	6	7	5	0	0	0	0	5
16	A7	53	0	0	0	9	27	9	0	0	0	0	8
17		1	0	0	0	1	0	0	0	0	0	0	0
18		3	0	0	0	2	0	1	0	0	0	0	0
19		1	0	0	0	1	0	0	0	0	0	0	0
20	A2	22	0	0	0	0	2	3	0	1	6	9	1
21		1	0	0	0	0	1	0	0	0	0	0	0
22		2	0	0	0	0	1	0	1	0	0	0	0
23		1	0	0	0	0	0	1	0	0	0	0	0
24		1	0	0	0	0	0	1	0	0	0	0	0
25		1	0	0	0	0	0	1	0	0	0	0	0
26		1	0	0	0	0	0	0	1	0	0	0	0
27		1	0	0	0	0	0	0	0	1	0	0	0
28		2	0	0	0	0	0	0	0	2	0	0	0
29		1	0	0	0	0	0	0	0	0	1	0	0
30		1	0	0	0	0	0	0	0	0	1	0	0
31		1	0	0	0	0	0	0	0	0	0	1	0
32		1	0	0	0	0	0	0	0	0	0	0	1
33		1	0	0	0	0	0	0	0	0	0	0	1
34		1	0	0	0	0	0	0	0	0	0	0	1
**total**		**1903**	**115**	**176**	**176**	**286**	**230**	**180**	**168**	**170**	**172**	**104**	**126**

Footnotes:
(a) haplotype numbers used only in Tables S12 and S13

Table S14 Haploytpe combinations in HGDP and other samples

population		haplotype combination				
HGDP code	name	A1+B6+C11	A5+B6+C11	A.other+B6+C11	B6+C11 total	C11 total
20	Orcadian	16	11	0	27	30
21	Adygei	21	11	0	32	34
22	Russian	26	20	0	46	47
24	Basque	26	17	1	44	48
25	French	19	31	1	51	55
27	Italian	15	6	2	23	24
28	Sardinian	17	33	1	51	55
29	Tuscan	8	5	1	14	16
34	Mozabite	20	24	0	44	50
36	Bedouin	33	49	2	84	88
37	Druze	24	48	6	78	84
38	Palestinian	30	50	4	84	90
50	Balochi	18	19	1	38	45
51	Brahui	24	22	1	47	49
52	Burusho	23	26	0	49	49
54	Hazara	23	6	0	29	29
56	Kalash	40	6	0	46	46
57	Makrani	23	17	0	40	43
58	Pathan (Pashtun)	18	21	1	40	41
59	Sindhi	20	17	1	38	39
86	Maya	1	3	0	4	4
430	BantuSouthAfrica	1	0	0	1	1
464	Mandenka	1	3	0	4	5
465	Yoruba	0	1	0	1	1
494	San	1	0	0	1	1
601	Han (a)	1	0	1	2	3
606	Dai (a)	0	1	0	1	1
607	Daur (a)	2	0	0	2	2
613	Oroqen (a)	3	0	0	3	3
617	Tu (a)	1	0	0	1	1
618	Xibo (a)	3	0	0	3	4
619	Yi (a)	1	0	0	1	1
622	Mongola (a)	3	0	0	3	3
629	Uygur	3	5	0	8	9
677	Cambodian (a)	0	1	0	1	1
699	Yakut	10	2	0	12	13
	Yemen	6	5	0	11	13
	Egypt	10	8	2	20	21
	Ethiopian Jews	3	6	0	9	12
	Ethiopian	7	11	0	18	21
	Saudi	21	15	1	37	37
	Morocco	5	10	1	16	18
	South Indian (mixed)	5	9	0	14	15
**East Asian subtotal (a)**		**14**	**2**	**1**	**18**	**20**
**total**		**532**	**519**	**27**	**1078**	**1152**

Table S15   Distribution of nucleotide substitutions in C and D regions

nucleotide change	fraction of total	
	hu:chimp (n=1227)	C11D4 (n=269)
A:G or G:A	0.306	0.342
T:C or C:T	0.355	0.346
A:T or T:A	0.077	0.056
C:G or G:C	0.082	0.063
A:C or C:A	0.103	0.097
T:G or G:T	0.077	0.097

File S1

Core region haplotypes defined by polymorphisms with MAF ≥ 1% in 1000 Genomes Project data

Available for download as a tab-delimited text file at http://www.g3journal.org/lookup/suppl/doi:10.1534/g3.113.007484/-/DC1

File S2

Haplotype combinations in regions B and C

Available for download as a tab-delimited text file at http://www.g3journal.org/lookup/suppl/doi:10.1534/g3.113.007484/-/DC1

File S3

Haplotype combinations in regions C and D

Available for download as a tab-delimited text file at http://www.g3journal.org/lookup/suppl/doi:10.1534/g3.113.007484/-/DC1

Files S4

Haplotype combinations in regions A and C

Available for download as a tab-delimited text file at http://www.g3journal.org/lookup/suppl/doi:10.1534/g3.113.007484/-/DC1
