# Supplementary material for: Molecular Phylogeography of a Human Autosomal Skin Color Locus Under Natural Selection
Source: G3 (Bethesda). 2013 Nov 1;3(11):2059–67. doi: 10.1534/g3.113.007484 (PMC3815065; doi:10.1534/g3.113.007484)
Supplement: Supporting Information [file supp_g3.113.007484_FigureS1.pdf]

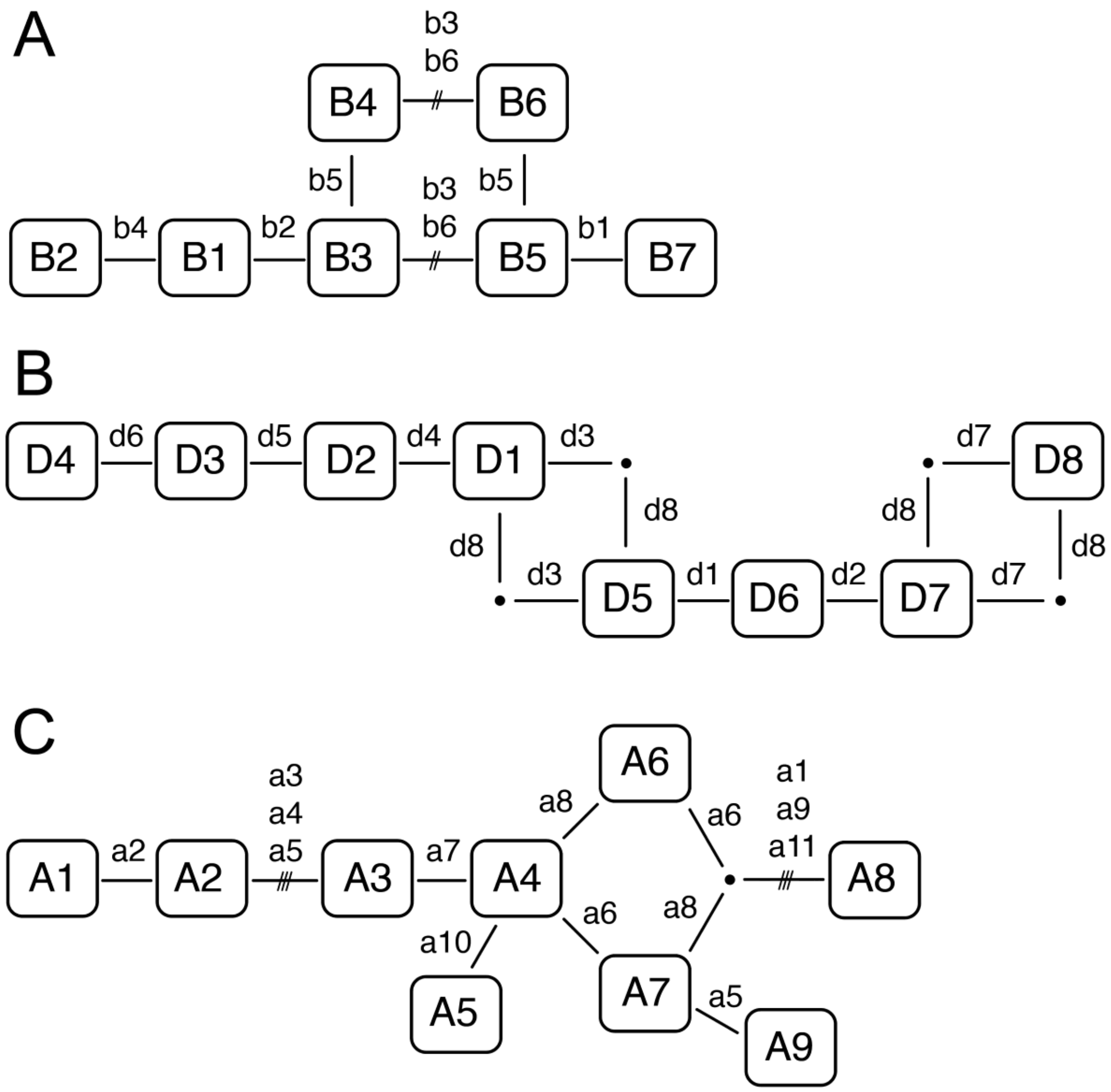

Figure S1 Common local haplotypes in regions outside core region
(A) Diagram showing haplotypes in B-subregion, defined using 6 SNPs.
(B) Haplotypes in D-subregion, defined using 8 SNPs.
(C) Haplotypes in A-subregion, defined using 11 SNPs.
The SNPs contributing to these haplotypes are identified in Table S2. Haplotypes carrying ancestral alleles of indicated SNPs are B1, D1, and A9. Full descriptions of haplotypes are in Tables S8-S13.
