# Supplementary material for: Molecular Phylogeography of a Human Autosomal Skin Color Locus Under Natural Selection
Source: G3 (Bethesda). 2013 Nov 1;3(11):2059–67. doi: 10.1534/g3.113.007484 (PMC3815065; doi:10.1534/g3.113.007484)
Supplement: Supporting Information [file supp_g3.113.007484_FigureS2.pdf]

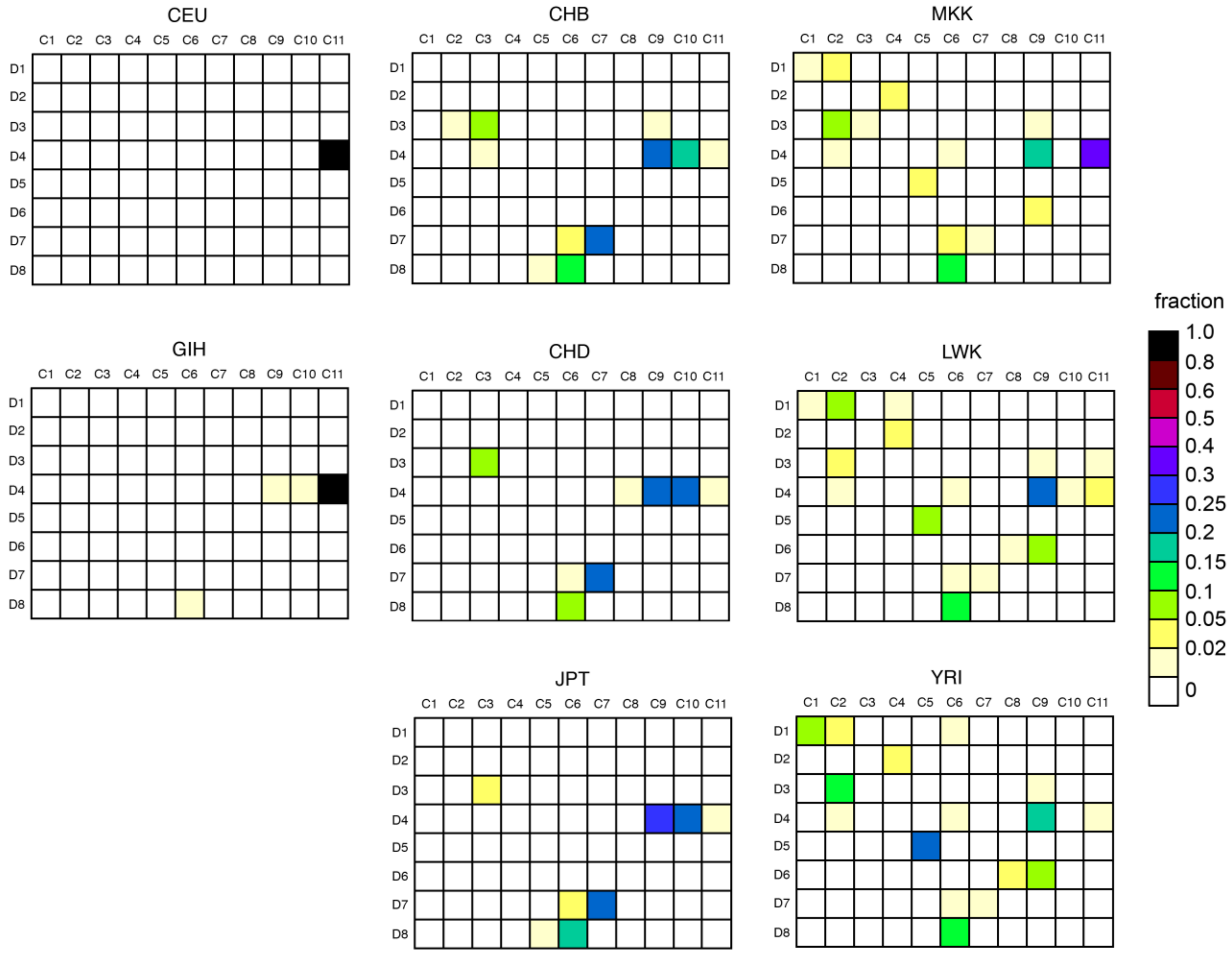

Figure S2 Relationships between local haplotypes in C and D subregions
For each HapMap population, distribution of combinations is shown as heat map (scale at right). Only common haplotypes are shown. Association of C5, C6, C7 and some African instances of C9 with D5-D8, while most members of the C9, C10, and C11 clusters associate with D4, indicates that a crossover between the C- and D-regions occurred in a chromosome that was ancestral to C10 and C11. Recurrent recombination between the core (C) region and D-region is apparent, as is the predominant association of C9, C10, and C11 with D4. Counts are shown in File S2.
