# Supplementary material for: Molecular Phylogeography of a Human Autosomal Skin Color Locus Under Natural Selection
Source: G3 (Bethesda). 2013 Nov 1;3(11):2059–67. doi: 10.1534/g3.113.007484 (PMC3815065; doi:10.1534/g3.113.007484)
Supplement: Supporting Information [file supp_g3.113.007484_FigureS3.pdf]

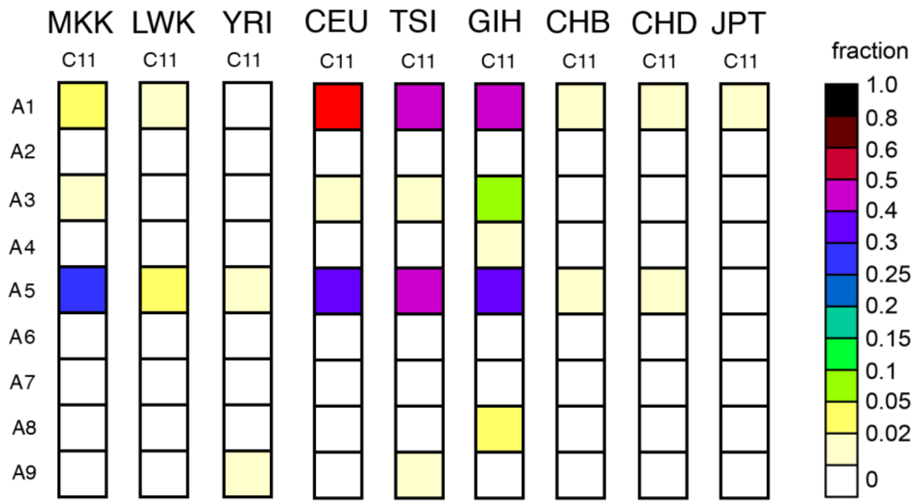

Figure S3 Association of A-region haplotypes with core haplotype C11
Scale (heat map, right) is relative to the total sample in each population, as in Figures 4 and S3. A1 and A5 are most commonly associated with C11. Counts are shown in File S3.
