# Supplementary material for: Molecular Phylogeography of a Human Autosomal Skin Color Locus Under Natural Selection
Source: G3 (Bethesda). 2013 Nov 1;3(11):2059–67. doi: 10.1534/g3.113.007484 (PMC3815065; doi:10.1534/g3.113.007484)
Supplement: Supporting Information [file supp_g3.113.007484_FigureS4.pdf]

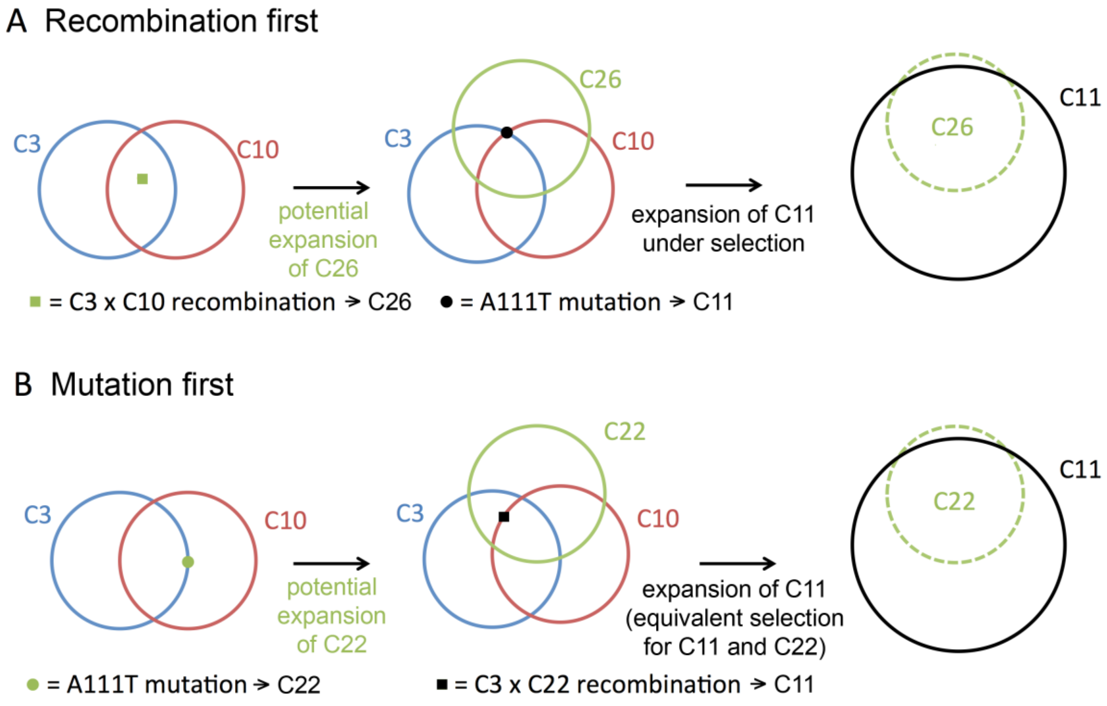

Figure S4 Evolutionary history of the C11 haplotype

Circles represent populations or geographical regions in which various haplotypes are found at successive time intervals. Dots indicate locations of events that created new haplotypes; dots on boundaries represent events that could have occurred in any of the adjoining regions. Diagrams show all potential patterns of co-occurrence, but are not intended to imply that all existed. Geographical regions at different time points may differ as a result of migration, selection, or genetic drift. **A.** Recombination first model. First diagram shows C3 and C10 with overlapping distributions. The green dot indicates recombination between C3 and C10 within their region of overlap to produce haplotype C26, a C11 precursor lacking the *A111T* mutation. At a subsequent time (second diagram), potential expansion of the range of C26 is shown. Creation of C11 by mutation in C26 at *A111T* (black dot) is independent of the distributions of C3 and C10. The third diagram represents the present. C11 expansion as a result of positive selection results in replacement of C26 within the region of fixation. C26 may potentially still exist outside the region in which C11 is fixed. **B.** Mutation first model. First diagram shows C3 and C10. Creation of C22 from C10 by the mutation *A111T* (green dot) is independent of the distribution of C3. At a subsequent time (second diagram), potential expansion or migration of C22 is shown. Recombination between C3 and C22 to produce C11 (black dot) is then independent of C10. The third diagram represents the present. C11 has expanded, while C22 has been reduced to a minor component. Since both haplotypes carry the *A111T* mutation

that is the target of selection, replacement in this model occurs via genetic drift or as a result of a small selective advantage (of unknown origin) of C11 over C22.
