# Supplementary material for: Molecular Phylogeography of a Human Autosomal Skin Color Locus Under Natural Selection
Source: G3 (Bethesda). 2013 Nov 1;3(11):2059–67. doi: 10.1534/g3.113.007484 (PMC3815065; doi:10.1534/g3.113.007484)
Supplement: Supporting Information [file supp_g3.113.007484_FigureS5.pdf]

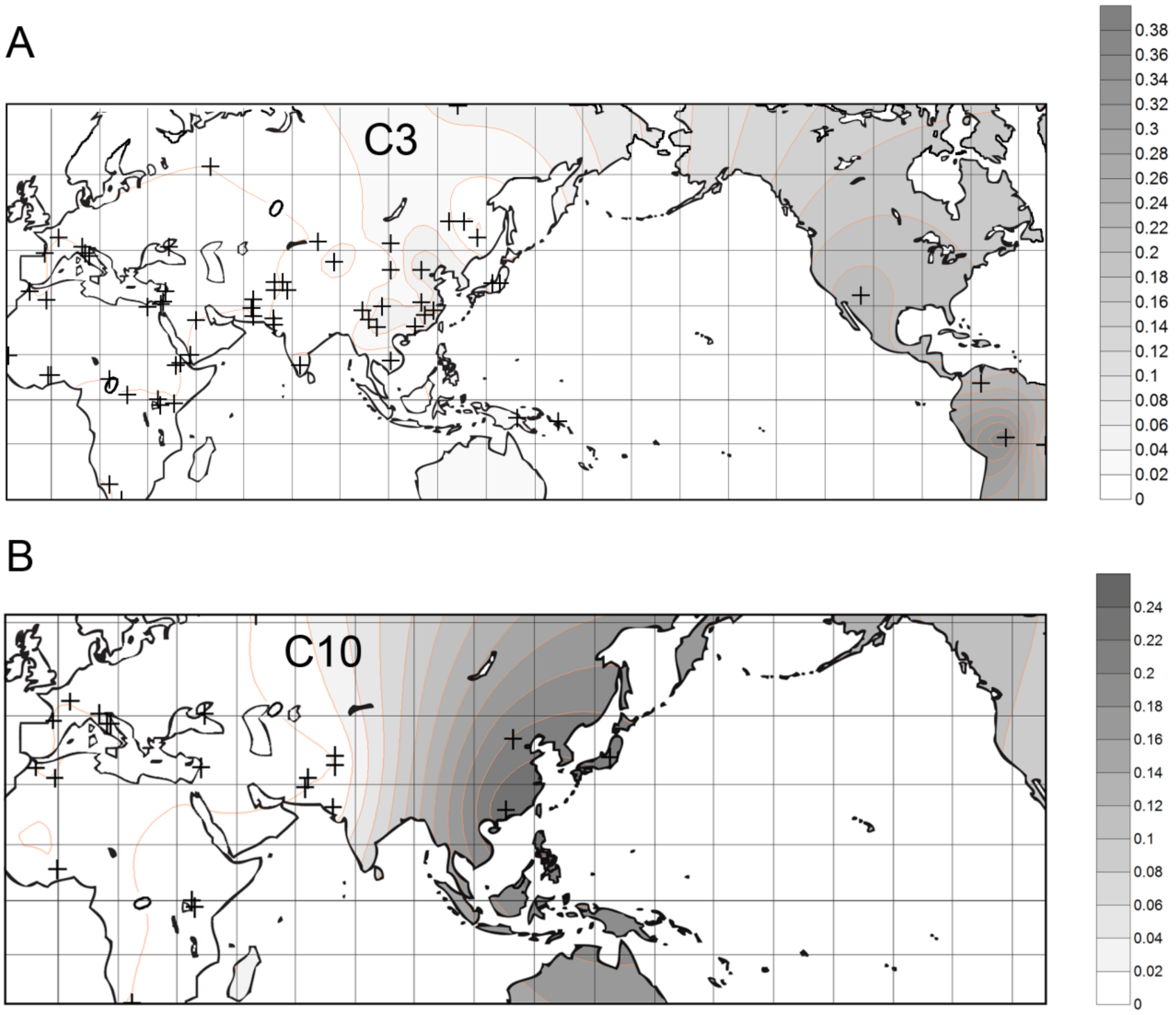

Figure S5 World distributions of haplotype C3 and C10
(A) Contour plot showing frequencies of haplotype C3
(B) Plot of frequency of haplotype C10
Scales are at right of each panel. Sampled populations are indicated (+).
