# Supplementary material for: Molecular Phylogeography of a Human Autosomal Skin Color Locus Under Natural Selection
Source: G3 (Bethesda). 2013 Nov 1;3(11):2059–67. doi: 10.1534/g3.113.007484 (PMC3815065; doi:10.1534/g3.113.007484)
Supplement: Supporting Information [file supp_g3.113.007484_TableS1.pdf]

Table S1 ***A111T*** **frequencies used to plot world distribution**

population name or code	sample size (2N)	*A111T* frequency	reference
Hezhen	18	0.000	A
Pima, Mexico	106	0.000	B
Koreans	106	0.000	B
Ticuna	130	0.046	B
Ami	80	0.000	B
Mbuti	78	0.000	B
Han	116	0.017	B
Japanese	98	0.020	B
Melanesian, Nasioi	46	0.000	B
Atayal	84	0.000	B
Karitiana	106	0.000	B
Micronesians	74	0.068	B
Bantu Kenya	24	0.000	A
Tujia	20	0.000	A
Yizu	20	0.000	A
Miaozu	20	0.000	A
Dai	20	0.000	A
Lahu	20	0.000	A
She	20	0.000	A
Naxi	20	0.000	A
Papuan	34	0.000	A
Orcadian	32	1.000	A
Columbian	26	0.000	A
Belorussian	18	0.944	C
Lithuanians	20	1.000	C
Biaka	138	0.007	B
Pima, Arizona	100	0.010	B
Danes	102	0.980	B
Surui	90	0.033	B
Hungarian	178	0.978	B
Japanese, JPT	172	0.012	D
Hakka	80	0.013	B
Yoruba	154	0.013	B
Yoruba, YRI	226	0.013	D
Irish	230	1.000	B
Finns	72	0.931	B
Maya, Yucatan	94	0.128	B
European Americans, CEU	116	1.000	D
Cheyenne	112	0.045	B
Han, CHB	168	0.030	D
Han	98	0.020	B
Lao Loum	238	0.034	B
Northern Italian	28	1.000	A

continued

Table S1 (continued)

population name or code	sample size (2N)	*A111T* frequency	reference
Ibo	96	0.042	B
Komi-Zyrian	92	0.913	B
Russians	66	1.000	B
Yakut	102	0.206	B
Mongola	20	0.150	A
Tu	20	0.050	A
Georgians	40	1.000	C
Europeans, Mixed	182	0.989	B
Russians	96	1.000	B
Bantu Southern	16	0.060	A
French Basque	48	1.000	A
Tuscan	16	1.000	A
Spaniards	115	0.987	B
African Americans	178	0.236	B
San	14	0.070	A
Chuvash	84	0.929	B
Hungarians	40	1.000	C
Cambodians, Khmer	50	0.080	B
Quechua	46	0.152	B
Zaramo	80	0.088	B
French	58	1.000	A
Romanians	32	0.938	C
Daur	20	0.100	A
Hausa	78	0.115	B
Chuvash	34	0.971	C
Sandawe	76	0.118	B
Khanty	98	0.806	B
Orogen	20	0.150	A
Mandenka	48	0.160	E
Adygei	108	0.982	B
Xibo	18	0.190	A
Uzbeks	30	0.600	C
Uygur	20	0.500	A
Spaniards	24	1.000	C
Chagga	90	0.267	B
Masai	40	0.275	B
Tamil	116	0.293	F
Lezgins	36	1.000	C
Armenians	38	1.000	C
Sardinian	56	0.980	A
Adygei	34	1.000	A
Cypriots	24	1.000	C
Turks	38	0.895	C
Syrians	32	1.000	C
Palestinian	102	0.990	A

continued

Table S1 (continued)

population name or code	sample size (2N)	*A111T* frequency	reference
Algerian	68	0.912	B
Kalash	50	1.000	A
Ethiopian Jewish	26	0.460	C
Lebanese	14	1.000	C
South Indian	38	0.470	C
Mozabite	60	0.870	A
Sinhalese	108	0.500	F
Druze	204	1.000	B
Jordanians	40	1.000	C
Hazara	48	0.610	A
Ethiopian	38	0.550	C
Jews, Ethiopian	64	0.578	B
Moroccans	96	0.979	B
Tunisian	194	0.923	B
Moroccan	20	0.900	C
Samaritans	78	1.000	B
Burusho	50	0.980	A
Libya	62	0.919	B
Egypt	24	0.880	C
Yemen	20	0.650	C
Iranians	40	0.925	C
Pathan (Pashtun)	48	0.960	A
Sindhi	50	0.830	A
Keralite	60	0.733	B
Bedouin	96	0.970	A
Brahui	50	1.000	A
Makrani	50	0.920	A
Balochi	50	0.980	A
Saudi	40	0.950	C
Maasai, MKK	286	0.329	D
Luhya, LWK	180	0.067	D
India, AA-C-IP5	36	0.110	G
India, AA-E-IP3	44	0.210	G
India, DR-C-IP2	40	0.020	G
India, DR-S-IP4	42	0.560	G
India, DR-S-LP2	44	0.440	G
India, DR-S-LP3	38	0.390	G
India, IE-E-IP1	44	0.270	G
India, IE-E-LP2	46	0.660	G
India, IE-E-LP4	44	0.610	G
India, IE-N-IP2	46	0.280	G
India, IE-N-LP1	42	0.630	G
India, IE-N-LP5	46	0.710	G
India, IE-N-LP9	46	0.560	G
India, IE-N-SP4	46	0.610	G

continued

Table S1 (concluded)

population name or code	sample size (2N)	*A111T* frequency	reference
India, IE-NE-IP1	44	0.260	G
India, IE-NE-LP1	44	0.480	G
India, IE-W-LP1	42	0.610	G
India, IE-W-LP2	42	0.600	G
India, IE-W-LP3	46	0.590	G
India, IE-W-LP4	44	0.690	G
India, TB-N-IP1	46	0.220	G
India, TB-N-SP1	46	0.360	G
India, TB-NE-LP1	44	0.220	G

References:
A, NORTON et al. 2007
B, CHEUNG et al. 2000, alfred.med.yale.edu/alfred/
C, BEHAR et al. 2010
D, ALTSHULER et al. 2010, http://hapmap.ncbi.nlm.nih.gov/
E, http://www.cephb.fr/en/cephdb/
F, SOEJIMA and KODA 2007
G, INDIAN GENOME VARIATION CONSORTIUM 2008, http://igvdb.res.in
