# Supplementary material for: Molecular Phylogeography of a Human Autosomal Skin Color Locus Under Natural Selection
Source: G3 (Bethesda). 2013 Nov 1;3(11):2059–67. doi: 10.1534/g3.113.007484 (PMC3815065; doi:10.1534/g3.113.007484)
Supplement: Supporting Information [file supp_g3.113.007484_TableS2.pdf]

Table S2 HapMap SNPs used for analysis

nickname	SNP_ ID	position (a)	alleles (b)	notes
		**A region**		
**nickname**	**SNP_ ID**	**position (a)**	**alleles (b)**	**notes**
a1	rs16960508	46108528	C/T	(e)
a2	rs1869453	46111620	G/A	(e)
a3	rs1426656	46114468	A/C	
a4	rs17340116	46114858	A/G	
a5	rs1453857	46116200	C/T	(e)
a6	rs2469594	46121836	G/T	
	rs2470104	46122159	T/C	(e)
a7	rs1025199	46126798	C/A	
a8	rs1365453	46129898	A/C	
a9	rs991877	46137615	T/C	(e)
a10	rs2433363	46139544	G/A	(e)
a11	rs16960535	46147951	T/C	
		**B region**		
**nickname**	**SNP_ ID**	**position**	**alleles**	**notes**
b1	rs16960541	46157395	G/T	
b2	rs2433360	46161326	A/C	
b3	rs2459392	46163796	G/A	
	rs10519163	46164805	C/T	(e)
b4	rs7180657	46168230	T/C	(e)
	rs2459383	46171162	G/A	(e)
b5	rs2250072	46172199	G/A	(e)
b6	rs2459385	46174380	C/A	(e)
		**C (core) region**		
**nickname**	**SNP_ ID**	**position**	**alleles**	**notes**
	rs12440301	46177216	G/A	(e)
	rs12441154	46178248	C/T	(f)
c1	rs1834640	46179457	G/A	(c, e)
	rs1559857	46184100	A/G	(e)
c2	rs2675345	46187491	G/A	(f)
c3	rs2469592	46189167	G/A	(e)
c4	rs2470101	46191159	C/T	(e)
c5	rs938505	46193187	C/T	(e)
c6	rs2433354	46202261	T/C	(e)
c7	rs2459391	46202360	G/A	(f)
c8	rs2433356	46203652	A/G	(e)
	rs16960620	46204191	A/G	(f, h)
c9	rs2675347	46205937	G/A	(e, g)
	rs2555364	46206678	C/G	(f, h)

continued

Table 2 (concluded)

c10	rs2675348	46208036	G/A	(e)
	rs8040016	46212039	C/T	(e)
	rs16960624	46212505	G/A	(e)
c11	rs1426654	46213776	G/A	(f, h)
c12	rs2470102	46220786	G/A	(f)
c13	rs16960631	46226209	A/G	(c, d, f)
c14	rs2675349	46232679	G/A	(f)
	rs3736482	46237809	T/C	(e)
	rs9652449	46240091	T/G	(e)
c15	rs3817315	46248438	T/C	(f)
c16	rs7163587	46255311	T/C	(f)
		**D region**		
**nickname**	**SNP_ ID**	**position**	**alleles**	**notes**
d1	rs2413886	46265512	T/G	(c, e)
d2	rs34722053	46268957	C/T	(c)
d3	rs8037482	46275588	A/G	(c, e)
d4	rs7170260	46288372	G/T	(c)
d5	rs1878186	46295692	C/T	(c, e)
d6	rs9920281	46301601	G/A	(e)
d7	rs8032941	46302144	T/C	(c)
d8	rs16960679	46304763	G/A	(c, d)

SNPs given nicknames (named SNPs) were genotyped and phased in all 11 HapMap phase 3 populations (release 27), except as otherwise noted. SNPs genotyped in HapMap phase 2 (release 21) are omitted here unless cited elsewhere.

Footnotes:
(a) NCBI B36 coordinates; for B37, add 2212708
(b) ancestral allele first
(c) named SNPs absent from TSI
(d) named SNPs absent from MEX
(e) present in HGDP
(f) core-region SNP absent from HGDP data
(g) absent from HapMap phase 2 release 21
(h) genotyped in GIARDINA *et al.* (2008)
