# Supplementary material for: Molecular Phylogeography of a Human Autosomal Skin Color Locus Under Natural Selection
Source: G3 (Bethesda). 2013 Nov 1;3(11):2059–67. doi: 10.1534/g3.113.007484 (PMC3815065; doi:10.1534/g3.113.007484)
Supplement: Supporting Information [file supp_g3.113.007484_TableS3.pdf]

Table S3 Description of core haplotypes determined using 16 SNPs

haplotype		SNP															
number (a)	name	c1	c2	c3	c4	c5	c6	c7	c8	c9	c10	c11	c12	c13	c14	c15	c16
18	C1	G	G	G	C	C	T	G	A	G	G	G	G	A	G	T	T
16	C2	G	G	G	C	T	T	G	A	G	G	G	G	A	G	T	T
17	C3	A	G	G	C	T	T	G	A	G	G	G	G	A	G	T	T
20	C4	G	G	G	T	C	T	G	A	G	G	G	G	A	G	T	T
19	C5	G	G	A	T	C	T	A	A	G	G	G	G	A	G	T	C
12	C6	G	G	A	T	C	T	A	G	G	G	G	G	A	G	T	C
22	C7	G	G	A	T	C	T	A	G	G	G	G	G	G	G	T	C
28	C8	G	G	A	T	C	C	A	G	A	G	G	G	A	A	C	C
8	C9	G	G	A	T	C	C	A	G	A	A	G	G	A	A	C	C
13	C10	G	A	A	T	C	C	A	G	A	A	G	A	A	A	C	C
1	C11	A	A	A	T	C	C	A	G	A	A	A	A	A	A	C	C
2	C25	A	A	A	T	T	C	A	A	A	A	A	A	A	A	C	C
3	C24	A	A	A	T	C	C	A	A	A	A	A	A	A	A	C	C
4	C23	A	A	A	T	C	C	A	G	A	A	A	A	A	A	T	C
5	(b)	A	A	A	T	T	C	G	A	G	A	A	G	A	G	C	C
7		A	G	A	T	C	C	A	G	A	A	A	A	A	A	C	C
9		G	G	A	T	C	C	A	G	A	A	A	A	A	A	C	C
10	(b)	A	A	A	T	C	C	A	G	A	A	A	A	A	G	T	C
15	(b)	A	G	G	C	T	C	G	A	A	A	A	A	A	A	C	C
25	C22	G	A	A	T	C	C	A	G	A	A	A	A	A	A	C	C
37		G	G	A	T	C	T	A	A	G	G	A	G	A	G	T	C
41		A	G	G	C	T	T	G	A	G	G	A	G	A	G	T	T
46		G	A	A	T	C	C	A	G	A	A	A	A	A	A	T	C
6	(b)	A	G	G	C	C	T	A	G	A	G	G	A	A	A	T	T
11	(b)	G	G	A	T	C	T	A	G	G	G	G	G	G	A	C	C
14	(b)	G	A	A	T	C	T	A	G	G	G	G	G	A	G	T	T
21	C20	G	G	A	T	C	C	A	G	A	A	G	G	A	G	T	C
23	C12	G	G	G	C	T	T	G	A	G	G	G	G	A	G	T	C
24		G	G	G	C	C	T	G	A	A	G	G	G	A	G	T	T
26	C17	G	G	A	T	C	T	G	G	G	G	G	G	A	G	T	C
27	C15	G	G	A	T	C	T	G	A	G	G	G	G	A	G	T	T
29	C16	G	G	A	T	C	T	G	A	G	G	G	G	A	G	T	C
30	C13	G	G	G	C	T	T	G	G	G	G	G	G	A	G	T	T
31		G	G	A	T	T	C	A	G	A	G	G	G	A	A	C	C
32		G	G	G	C	C	T	G	G	G	G	G	G	A	G	T	C
33	C14	G	G	G	C	T	T	G	G	G	G	G	G	A	G	T	C
34		G	G	G	T	C	C	A	G	A	A	G	G	A	A	C	C
35		G	G	A	T	C	T	A	G	A	G	G	G	A	G	T	C
36		G	G	A	T	C	T	A	G	A	G	G	G	G	G	T	C
38		G	G	A	T	C	C	A	G	A	A	G	G	G	G	C	C
39	C18	G	G	A	T	C	C	A	G	A	A	G	G	A	A	T	C

continued

Table S3 (concluded)

haplotype		SNP															
**number (a)**	**name**	**c1**	**c2**	**c3**	**c4**	**c5**	**c6**	**c7**	**c7**	**c9**	**c10**	**c11**	**c12**	**c13**	**c14**	**c15**	**c16**
40		G	G	A	T	C	T	A	A	G	A	G	G	A	G	T	C
42	C21	G	G	A	T	C	C	A	G	A	A	G	A	A	A	C	C
43		G	G	A	T	C	C	A	G	A	G	G	G	A	G	C	T
44		A	G	A	T	C	T	A	G	G	G	G	G	A	G	T	C
45		G	G	A	T	C	T	A	G	G	G	G	G	A	A	C	C
47		G	A	A	T	C	C	A	G	A	A	G	G	G	A	T	C
48		A	G	G	C	T	T	G	A	A	G	G	G	A	G	T	T
49		G	A	A	T	C	C	A	A	A	A	G	A	A	A	C	C
50	C26	A	A	A	T	C	C	A	G	A	A	G	A	A	A	C	C
51	C19	G	G	A	T	C	C	A	G	A	A	G	G	A	G	C	C
52		G	G	A	T	C	T	A	G	G	G	G	G	A	A	T	C

Footnotes:
(a) Haplotype numbers used only in Tables S3 and S4.
(b) Three misphased pairs are, after correction:
5+6: C11+C2/C3
10+11: C11+C7
14+15: C11+C2
