# Supplementary material for: Molecular Phylogeography of a Human Autosomal Skin Color Locus Under Natural Selection
Source: G3 (Bethesda). 2013 Nov 1;3(11):2059–67. doi: 10.1534/g3.113.007484 (PMC3815065; doi:10.1534/g3.113.007484)
Supplement: Supporting Information [file supp_g3.113.007484_TableS4.pdf]

Table S4 Population distribution of core haplotypes determined using 16 SNPs

haplotype			population										
number (a)	name	total	CEU	TSI	GIH	MKK	YRI	LWK	CHB	CHD	JPT	MEX	ASW
18	C1	25	0	0	0	7	13	4	0	0	0	0	1
16	C2	125	0	0	0	38	38	26	1	0	0	1	21
17	C3	45	0	0	0	3	0	0	14	14	5	9	0
20	C4	30	0	0	0	6	7	14	0	0	0	0	3
19	C5	98	0	0	0	13	50	14	1	0	1	0	19
12	C6	245	0	0	1	52	42	33	34	21	35	7	20
22	C7	122	0	0	0	1	3	2	40	39	36	0	1
28	C8	10	0	0	0	0	5	1	0	1	0	0	3
8	C9	365	0	0	3	65	61	63	36	44	51	15	27
13	C10	123	0	0	1	0	0	1	34	41	37	9	0
1	C11	638	109	172	164	92	3	9	3	3	1	60	22
2	C25	2	2	0	0	0	0	0	0	0	0	0	0
3	C24	3	2	0	0	0	0	1	0	0	0	0	0
4	C23	5	2	1	0	0	0	0	1	0	0	1	0
5		1	0	1	0	0	0	0	0	0	0	0	0
7		2	0	1	0	0	0	0	0	0	0	1	0
9		1	0	0	1	0	0	0	0	0	0	0	0
10		2	0	0	2	0	0	0	0	0	0	0	0
15		1	0	0	1	0	0	0	0	0	0	0	0
25	C22	4	0	0	0	2	0	1	1	0	0	0	0
37		1	0	0	0	0	0	1	0	0	0	0	0
41		1	0	0	0	0	0	0	0	1	0	0	0
46		1	0	0	0	0	0	0	0	0	1	0	0
6		1	0	1	0	0	0	0	0	0	0	0	0
11		2	0	0	2	0	0	0	0	0	0	0	0
14		1	0	0	1	0	0	0	0	0	0	0	0
21	C20	5	0	0	0	4	1	0	0	0	0	0	0
23	C12	2	0	0	0	2	0	0	0	0	0	0	0
24		1	0	0	0	1	0	0	0	0	0	0	0
26	C17	8	0	0	0	0	1	5	0	0	0	0	2
27	C15	4	0	0	0	0	2	0	0	0	0	0	2
29	C16	1	0	0	0	0	1	0	0	0	0	0	0
30	C13	1	0	0	0	0	1	0	0	0	0	0	0
31		1	0	0	0	0	1	0	0	0	0	0	0
32		1	0	0	0	0	1	0	0	0	0	0	0
33	C14	3	0	0	0	0	0	2	0	0	0	0	1
34		1	0	0	0	0	0	1	0	0	0	0	0
35		1	0	0	0	0	0	1	0	0	0	0	0
36		1	0	0	0	0	0	1	0	0	0	0	0
38		2	0	0	0	0	0	0	1	1	0	0	0
39	C18	2	0	0	0	0	0	0	1	0	1	0	0

continued

Table S4 (concluded)

haplotype		total	population										
number (a)	name		CEU	TSI	GIH	MKK	YRI	LWK	CHB	CHD	JPT	MEX	ASW
40		2	0	0	0	0	0	0	1	1	0	0	0
42	C21	2	0	0	0	0	0	0	0	2	0	0	0
43		1	0	0	0	0	0	0	0	1	0	0	0
44		1	0	0	0	0	0	0	0	1	0	0	0
45		1	0	0	0	0	0	0	0	0	1	0	0
47		1	0	0	0	0	0	0	0	0	1	0	0
48		1	0	0	0	0	0	0	0	0	1	0	0
49		1	0	0	0	0	0	0	0	0	1	0	0
50	C26	1	0	0	0	0	0	0	0	0	0	1	0
51	C19	3	0	0	0	0	0	0	0	0	0	0	3
52		1	0	0	0	0	0	0	0	0	0	0	1
**total**		**1903**	**115**	**176**	**176**	**286**	**230**	**180**	**168**	**170**	**172**	**104**	**126**

Footnotes:
(a) Haplotype numbers used only in Tables S3 and S4.
