# Supplementary material for: Molecular Phylogeography of a Human Autosomal Skin Color Locus Under Natural Selection
Source: G3 (Bethesda). 2013 Nov 1;3(11):2059–67. doi: 10.1534/g3.113.007484 (PMC3815065; doi:10.1534/g3.113.007484)
Supplement: Supporting Information [file supp_g3.113.007484_TableS5.pdf]

Table S5 Core haplotypes in HGDP and other samples

haplotype		SNP (a)															
number (b)	name (c)	c1	'857	c3	c4	c5	c6	c8	c9	c10	'016	'624	c11	'482	'449	HGDP count	other (d)
14	C1	G	A	G	C	C	T	A	G	G	C	G	G	T	T	9	
13	C1	G	A	G	C	C	T	A	G	G	C	G	G	T	G	2	
12	C1	G	A	G	C	C	T	A	G	G	C	G	G	C	G	1	
16	C2	G	A	G	C	T	T	A	G	G	C	G	G	T	G	28	7
18	C2	G	A	G	C	T	T	A	G	G	T	G	G	T	T	6	6
17	C2	G	A	G	C	T	T	A	G	G	C	G	G	T	T	3	1
15	C2	G	A	G	C	T	T	A	G	G	C	G	G	C	G	1	
24	C2	G	A	G	C	T	T	A	G	G	T	G	G	C	G		1
23	C3	A	A	G	C	T	T	A	G	G	T	G	G	T	T	80	1
11	C4	G	A	G	T	C	T	A	G	G	C	G	G	T	T	15	
10	C4	G	A	G	T	C	T	A	G	G	C	G	G	T	G	3	
7	C5	G	A	A	T	C	T	A	G	G	C	G	G	C	G	20	1
8	C5	G	A	A	T	C	T	A	G	G	C	A	G	C	G	19	1
6	C6/ C7	G	A	A	T	C	T	G	G	G	C	G	G	C	G	279	20
2	C8	G	G	A	T	C	C	G	A	G	C	G	G	C	G	4	
1a	C9/ C10	G	G	A	T	C	C	G	A	A	C	G	G	C	G	374	28
5	C9/ C10	G	A	A	T	C	C	G	A	A	C	G	G	C	G	5	
19a	C26	A	G	A	T	C	C	G	A	A	C	G	G	C	G	2	
19d	C11	A	G	A	T	C	C	G	A	A	C	G	A	C	G	1005	133
20	C11	A	A	A	T	C	C	G	A	A	C	G	A	C	G	4	2
1d	C22	G	G	A	T	C	C	G	A	A	C	G	A	C	G	14	
4		G	G	A	T	C	C	G	G	G	C	G	G	C	G	2	
3		G	G	A	T	C	C	G	G	A	C	G	G	C	G	1	
9		G	A	G	T	C	T	G	G	G	C	G	G	C	G	1	2
21		A	A	A	T	C	T	G	G	G	C	G	G	C	G	1	
22		A	A	G	C	T	C	G	A	A	C	G	G	C	G	1	
25		G	A	A	T	C	C	G	A	A	C	G	A	C	G		1
**total**																**1880**	**204**

Footnotes:

(a) SNPs shared with HapMap phase 3 data are identified by nickname; others by last three digits of rs# (Table S2)
(b) haplotype designations specific to this table
(c) equivalents classified using 11 SNPs
(d) data from BEHAR et al. 2010
