# Supplementary material for: Molecular Phylogeography of a Human Autosomal Skin Color Locus Under Natural Selection
Source: G3 (Bethesda). 2013 Nov 1;3(11):2059–67. doi: 10.1534/g3.113.007484 (PMC3815065; doi:10.1534/g3.113.007484)
Supplement: Supporting Information [file supp_g3.113.007484_TableS6.pdf]

Table S6 Distribution of core haplotypes in HGDP and other samples

HGDP code	population name	haplotype												total
		C1	C2	C3	C4	C5	C6/ C7	C8	C9/ C10	C26	C11	C22	others	
20	Orcadian										30			30
21	Adygei										34			34
22	Russian										47	3		50
24	Basque										48			48
25	French										55	1		56
27	Italian										24			24
28	Sardinian				1						55			56
29	Tuscan										16			16
34	Mozabite		2			3	2	1			50			58
36	Bedouin		2				1				88	1		92
37	Druze										84			84
38	Palestinian						1		1		90			92
50	Balochi		1								45	2		48
51	Brahui										49	1		50
52	Burusho								1		49			50
54	Hazara		1				6		8		29			44
56	Kalash										46			46
57	Makrani		1				1		2		43	3		50
58	Pathan (Pashtun)						2				41	1		44
59	Sindhi						5		3		39	1		48
71	Melanesian						16		4					20
75	Papuan			2			25		7					34
81	Colombian			5			1		8					14
82	Karitiana			15					13					28
83	Surui			5					11					16
86	Maya			12			6		19		4		1	42
87	Pima			10					18					28
430	Bantu SouthAfrica		4			4	2		5		1			16
441	BantuKenya	1	3		2	3	7		6					22
464	Mandenka	3	2		1	14	12		7		5			44
465	Yoruba	1	3		1	11	9	2	14		1			42
488	BiakaPygmy	3	5		6	2	5		21					42
489	MbutiPygmy	3	5		7	2	1		7				1	26
494	San	1	8								1			10

continued

Table S6 (concluded)

HGDP code	population name	haplotype												total
		C1	C2	C3	C4	C5	C6/ C7	C8	C9/ C10	C26	C11	C22	others	
601	Han			5			22		39	1	1			68
602	Han-NChina			3			7		10					20
606	Dai		1	2			9		8					20
607	Daur			1			7		8		2			18
608	Hezhen						7		8				1	16
611	Lahu			1			8		7					16
612	Miao			3			4		12				1	20
613	Oroqen						6		9		3			18
615	She			3			7		9				1	20
616	Tujia			1			10		9					20
617	Tu						7		12		1			20
618	Xibo						5		9	1	3			18
619	Yi			2			5		13					20
622	Mongola			1			11		5		3			20
625	Naxi						8		8					16
629	Uygur			1			7		2		9	1		20
677	Cambodian						10		8		1		1	20
684	Japanese			4			21	1	30					56
699	Yakut			4			16		18		12			50
	Moroccan					1	1				18			20
	Egyptian		1						2		21			24
	Ethiopian (mixed)		6				4		7		21			38
	Ethiopian Jewish		5				1		6		12		2	26
	Saudi						1		1		37		1	40
	Yemeni		1			1	3		2		13			20
	South Indian (mixed)		2	1			10		10		15			38
**subtypes**		**3**	**5**	**1**	**2**	**2**	**1**	**1**	**2**	**1**	**2**	**1**	**6**	
**total**		**12**	**53**	**81**	**18**	**41**	**299**	**4**	**407**	**2**	**1146**	**14**	**9**	**2086**
**most common subtype**		**9**	**35**	**81**	**15**	**21**	**299**	**4**	**402**	**2**	**1138**	**14**	**4**	
