# Supplementary material for: Molecular Phylogeography of a Human Autosomal Skin Color Locus Under Natural Selection
Source: G3 (Bethesda). 2013 Nov 1;3(11):2059–67. doi: 10.1534/g3.113.007484 (PMC3815065; doi:10.1534/g3.113.007484)
Supplement: Supporting Information [file supp_g3.113.007484_TableS7.pdf]

Table S7   Apparent fragmentation of core-region haplotypes varies with SNP frequency.

polymorphism cutoff frequency	total polymorphisms	total haplotypes	common haplotypes	fraction in common haplotypes
0.1	24	104	15	0.92
0.05	73	187	21	0.85
0.02	109	215	21	0.83
0.01	156	291	34	0.78
0.005	239	349	39	0.72
0.0025	287	403	34	0.64
0.0009	504	546	28	0.54
all SNPs	744	653	26	0.52
all (0.045%)	767	716	25	0.48

Common haplotypes are defined as those with frequencies > 0.5% in combined sample. Values here are not corrected for undercounting of rare SNPs.
