# Supplementary material for: Molecular Phylogeography of a Human Autosomal Skin Color Locus Under Natural Selection
Source: G3 (Bethesda). 2013 Nov 1;3(11):2059–67. doi: 10.1534/g3.113.007484 (PMC3815065; doi:10.1534/g3.113.007484)
Supplement: Supporting Information [file supp_g3.113.007484_TableS8.pdf]

Table S8 Description of B region haplotypes

haplotype			SNP (a)					
number (b)	name		b1	b2	b3	b4	b5	b6
		ancestral state	G	A	G	T	G	C
1	B6		G	C	A	T	A	A
2	B7		T	C	A	T	G	A
3			G	C	G	C	G	C
4	B2		G	A	G	C	G	C
5	B5		G	C	A	T	G	A
6	B3		G	C	G	T	G	C
7			T	C	A	T	G	C
8	B1		G	A	G	T	G	C
9			T	A	G	T	G	C
10	B4		G	C	G	T	A	C
11			G	A	A	T	A	A
12			T	C	A	T	A	A
13			T	C	G	T	G	A
14			G	C	A	T	G	C
15			G	A	A	T	G	A
16			G	C	A	T	A	C
17			T	A	G	C	G	C
18			T	C	G	T	G	C
19			G	C	G	T	G	A
20			G	C	A	C	G	C
21			G	A	G	C	G	A

Footnotes:
(a) SNPs identified by nickname (Table S2)
(b) Haplotype numbers used only in Tables S8 and S9
