# Supplementary material for: Molecular Phylogeography of a Human Autosomal Skin Color Locus Under Natural Selection
Source: G3 (Bethesda). 2013 Nov 1;3(11):2059–67. doi: 10.1534/g3.113.007484 (PMC3815065; doi:10.1534/g3.113.007484)
Supplement: Supporting Information [file supp_g3.113.007484_TableS9.pdf]

Table S9 Population distribution of B region haplotypes

haplotype			population										
**number (a)**	**name**	**total**	**CEU**	**TSI**	**GIH**	**MKK**	**YRI**	**LWK**	**CHB**	**CHD**	**JPT**	**MEX**	**ASW**
1	B6	673	109	167	151	100	2	9	23	20	12	65	15
2	B7	238	4	6	2	65	33	20	21	27	28	14	18
3		4	2	1	0	0	0	0	0	0	0	0	1
4	B2	511	0	2	13	59	106	76	59	61	68	18	49
5	B5	238	0	0	4	37	64	49	18	20	17	3	26
6	B3	137	0	0	5	0	3	0	43	38	42	3	3
7		1	0	0	1	0	0	0	0	0	0	0	0
8	B1	51	0	0	0	14	12	13	1	0	0	0	11
9		5	0	0	0	5	0	0	0	0	0	0	0
10	B4	20	0	0	0	3	10	5	0	0	0	0	2
11		2	0	0	0	2	0	0	0	0	0	0	0
12		3	0	0	0	1	0	0	0	1	0	1	0
13		3	0	0	0	0	0	3	0	0	0	0	0
14		2	0	0	0	0	0	1	0	1	0	0	0
15		2	0	0	0	0	0	2	0	0	0	0	0
16		1	0	0	0	0	0	1	0	0	0	0	0
17		3	0	0	0	0	0	1	1	0	1	0	0
18		2	0	0	0	0	0	0	1	0	1	0	0
19		5	0	0	0	0	0	0	1	1	3	0	0
20		1	0	0	0	0	0	0	0	1	0	0	0
21		1	0	0	0	0	0	0	0	0	0	0	1
**total**		**1903**	**115**	**176**	**176**	**286**	**230**	**180**	**168**	**170**	**172**	**104**	**126**

Footnotes:
(a) haplotype numbers used only in Tables S8 and S9
