# Supplementary material for: Molecular Phylogeography of a Human Autosomal Skin Color Locus Under Natural Selection
Source: G3 (Bethesda). 2013 Nov 1;3(11):2059–67. doi: 10.1534/g3.113.007484 (PMC3815065; doi:10.1534/g3.113.007484)
Supplement: Supporting Information [file supp_g3.113.007484_TableS10.pdf]

Table S10 Description of D region haplotypes

haplotype			SNP (a)							
number (b)	name		d1	d2	d3	d4	d5	d6	d7	d8
		ancestral state	T	C	A	G	C	G	T	G
1	D4		T	C	A	T	T	A	T	G
2	D3		T	C	A	T	T	G	T	G
12	D2		T	C	A	T	C	G	T	G
5	D1		T	C	A	G	C	G	T	G
11	D5		T	C	G	G	C	G	T	A
10	D6		G	C	G	G	C	G	T	A
3	D7		G	T	G	G	C	G	T	A
4	D8		G	T	G	G	C	G	C	G
9			G	T	G	G	C	G	T	G
16			G	T	G	G	C	A	T	G
14			G	C	G	G	C	G	T	G
19			T	C	A	T	C	G	T	A
8			T	C	A	G	C	G	T	A
15			G	C	G	G	C	G	C	G
22			T	C	A	T	T	G	C	G
18			T	C	G	G	C	G	T	G
6			T	C	A	G	C	G	C	G
21			T	C	A	T	T	G	T	A
23			T	C	A	T	T	A	T	A
26			T	C	A	G	T	A	T	G
17			T	T	G	G	C	G	T	G
13			T	C	A	T	C	G	C	G
7			T	C	A	G	C	A	T	G
24			G	T	G	G	T	G	T	A
20			T	C	G	G	C	A	T	G
25			T	C	A	T	T	A	C	G
27			G	T	A	T	T	G	T	G
28			G	C	G	G	T	A	T	G
29			G	T	A	T	T	A	T	G
30			G	T	G	G	C	G	C	A
31			G	C	A	T	T	G	C	G
32			T	T	G	G	C	G	C	G
33			T	C	G	G	C	G	C	G
**total**										

Footnotes:

(a) SNPs identified by nickname (Table S2)

(b) Haplotype numbers used only in Tables S10 and S11
