# Supplementary material for: Molecular Phylogeography of a Human Autosomal Skin Color Locus Under Natural Selection
Source: G3 (Bethesda). 2013 Nov 1;3(11):2059–67. doi: 10.1534/g3.113.007484 (PMC3815065; doi:10.1534/g3.113.007484)
Supplement: Supporting Information [file supp_g3.113.007484_TableS11.pdf]

Table S11 Population distribution of D region haplotypes

haplotype			population										
number (a)	name	total	CEU	TSI	GIH	MKK	YRI	LWK	CHB	CHD	JPT	MEX	ASW
1	D4	1080	115	173	172	139	47	52	73	90	92	86	41
2	D3	122	0	3	0	23	27	10	15	15	6	11	12
12	D2	25	0	0	0	6	8	9	0	0	0	0	2
5	D1	67	0	0	1	15	20	17	0	0	0	0	14
11	D5	89	0	0	0	7	50	15	0	0	0	0	17
10	D6	59	0	0	0	14	18	19	0	0	0	0	8
3	D7	154	0	0	2	9	5	5	49	41	41	0	2
4	D8	192	0	0	1	32	34	30	23	17	30	7	18
9		13	0	0	0	10	1	0	1	1	0	0	0
16		13	0	0	0	3	3	4	2	1	0	0	0
14		10	0	0	0	3	2	3	0	0	1	0	1
19		9	0	0	0	1	3	5	0	0	0	0	0
8		8	0	0	0	4	1	3	0	0	0	0	0
15		7	0	0	0	2	2	2	0	0	0	0	1
22		7	0	0	0	0	5	0	0	1	0	0	1
18		7	0	0	0	5	1	0	0	0	0	0	1
6		7	0	0	0	5	1	1	0	0	0	0	0
21		5	0	0	0	0	1	2	0	0	0	0	2
23		5	0	0	0	0	1	2	1	0	0	0	1
26		4	0	0	0	0	0	0	1	2	1	0	0
17		4	0	0	0	4	0	0	0	0	0	0	0
13		3	0	0	0	1	0	0	1	0	0	0	1
7		2	0	0	0	2	0	0	0	0	0	0	0
24		2	0	0	0	0	0	1	0	0	0	0	1
20		1	0	0	0	1	0	0	0	0	0	0	0
25		1	0	0	0	0	0	0	1	0	0	0	0
27		1	0	0	0	0	0	0	1	0	0	0	0
28		1	0	0	0	0	0	0	0	1	0	0	0
29		1	0	0	0	0	0	0	0	1	0	0	0
30		1	0	0	0	0	0	0	0	0	1	0	0
31		1	0	0	0	0	0	0	0	0	0	0	1
32		1	0	0	0	0	0	0	0	0	0	0	1
33		1	0	0	0	0	0	0	0	0	0	0	1
**total**		**1903**	**115**	**176**	**176**	**286**	**230**	**180**	**168**	**170**	**172**	**104**	**126**

Footnotes:
(a) haplotype numbers used only in Tables S10 and S11
