# Supplementary material for: Molecular Phylogeography of a Human Autosomal Skin Color Locus Under Natural Selection
Source: G3 (Bethesda). 2013 Nov 1;3(11):2059–67. doi: 10.1534/g3.113.007484 (PMC3815065; doi:10.1534/g3.113.007484)
Supplement: Supporting Information [file supp_g3.113.007484_TableS12.pdf]

Table S12   Description of A region haplotypes

haplotype			SNP (a)										
**number (b)**	**name**		**a1**	**a2**	**a3**	**a4**	**a5**	**a6**	**a7**	**a8**	**a9**	**a10**	**a11**
		**ancestral state**	**C**	**G**	**A**	**A**	**C**	**G**	**C**	**A**	**T**	**G**	**T**
1	A1		C	A	C	G	C	T	A	A	T	G	T
2	A5		C	G	A	A	T	T	C	A	T	A	T
3	A3		C	G	A	A	T	T	A	A	T	G	T
4			C	A	C	G	T	T	A	A	T	G	T
5			T	G	A	A	T	G	C	C	T	G	T
6			C	G	A	A	T	T	C	A	C	A	C
7	A9		C	G	A	A	C	G	C	A	T	G	T
8	A8		T	G	A	A	T	G	C	C	C	G	C
9			T	G	A	A	T	T	A	A	T	G	T
10	A4		C	G	A	A	T	T	C	A	T	G	T
11			C	G	A	A	C	G	C	A	T	A	T
12			T	G	A	A	T	G	A	A	T	G	T
13			C	A	C	G	C	T	C	A	T	A	T
14			C	G	A	A	T	G	C	A	T	A	T
15	A6		C	G	A	A	T	T	C	C	T	G	T
16	A7		C	G	A	A	T	G	C	A	T	G	T
17			C	G	C	G	C	T	C	A	T	G	T
18			C	G	A	A	T	T	C	C	C	G	C
19			C	G	C	G	C	T	C	A	T	A	T
20	A2		C	G	C	G	C	T	A	A	T	G	T
21			C	A	A	A	C	G	C	A	T	G	T
22			C	A	C	G	C	T	A	A	T	A	T
23			C	G	A	A	T	T	A	A	T	A	T
24			C	G	A	G	C	G	C	A	T	G	T
25			C	A	A	A	T	G	C	A	T	G	T
26			C	A	A	A	T	T	A	A	T	G	T
27			C	G	A	G	C	T	C	A	T	G	T
28			C	A	C	G	C	T	A	A	T	G	C
29			C	A	C	A	C	T	A	A	T	G	T
30			C	A	A	A	T	T	A	A	T	A	T
31			T	A	A	A	T	G	C	C	C	G	C
32			T	G	A	A	T	G	C	A	T	G	C
33			C	G	A	A	T	T	C	C	C	G	T
34			C	G	A	A	T	T	C	C	T	A	T
**total**													

Footnotes:
(a) SNPs identified by nickname (Table S2)
(b) Haplotype numbers used only in Tables S12 and S13
