# Supplementary material for: Molecular Phylogeography of a Human Autosomal Skin Color Locus Under Natural Selection
Source: G3 (Bethesda). 2013 Nov 1;3(11):2059–67. doi: 10.1534/g3.113.007484 (PMC3815065; doi:10.1534/g3.113.007484)
Supplement: Supporting Information [file supp_g3.113.007484_TableS13.pdf]

Table S13 Population distribution of A region haplotypes

haplotype			population										
number (a)	name	total	CEU	TSI	GIH	MKK	YRI	LWK	CHB	CHD	JPT	MEX	ASW
1	A1	660	73	85	93	40	41	22	74	77	79	39	37
2	A5	597	41	83	64	158	57	61	28	26	11	38	30
3	A3	116	1	3	10	3	0	1	33	26	37	2	0
4		3	0	2	0	0	0	0	0	0	0	1	0
5		1	0	1	0	0	0	0	0	0	0	0	0
6		1	0	1	0	0	0	0	0	0	0	0	0
7	A9	225	0	1	0	35	86	61	0	0	0	1	41
8	A8	128	0	0	5	2	2	4	29	36	37	13	0
9		1	0	0	1	0	0	0	0	0	0	0	0
10	A4	25	0	0	1	10	4	7	2	0	0	0	1
11		6	0	0	1	1	2	2	0	0	0	0	0
12		2	0	0	1	0	0	0	0	1	0	0	0
13		17	0	0	0	16	0	1	0	0	0	0	0
14		2	0	0	0	2	0	0	0	0	0	0	0
15	A6	23	0	0	0	6	7	5	0	0	0	0	5
16	A7	53	0	0	0	9	27	9	0	0	0	0	8
17		1	0	0	0	1	0	0	0	0	0	0	0
18		3	0	0	0	2	0	1	0	0	0	0	0
19		1	0	0	0	1	0	0	0	0	0	0	0
20	A2	22	0	0	0	0	2	3	0	1	6	9	1
21		1	0	0	0	0	1	0	0	0	0	0	0
22		2	0	0	0	0	1	0	1	0	0	0	0
23		1	0	0	0	0	0	1	0	0	0	0	0
24		1	0	0	0	0	0	1	0	0	0	0	0
25		1	0	0	0	0	0	1	0	0	0	0	0
26		1	0	0	0	0	0	0	1	0	0	0	0
27		1	0	0	0	0	0	0	0	1	0	0	0
28		2	0	0	0	0	0	0	0	2	0	0	0
29		1	0	0	0	0	0	0	0	0	1	0	0
30		1	0	0	0	0	0	0	0	0	1	0	0
31		1	0	0	0	0	0	0	0	0	0	1	0
32		1	0	0	0	0	0	0	0	0	0	0	1
33		1	0	0	0	0	0	0	0	0	0	0	1
34		1	0	0	0	0	0	0	0	0	0	0	1
**total**		**1903**	**115**	**176**	**176**	**286**	**230**	**180**	**168**	**170**	**172**	**104**	**126**

Footnotes:
(a) haplotype numbers used only in Tables S12 and S13
