# Supplementary material for: Molecular Phylogeography of a Human Autosomal Skin Color Locus Under Natural Selection
Source: G3 (Bethesda). 2013 Nov 1;3(11):2059–67. doi: 10.1534/g3.113.007484 (PMC3815065; doi:10.1534/g3.113.007484)
Supplement: Supporting Information [file supp_g3.113.007484_TableS14.pdf]

Table S14 Haploytpe combinations in HGDP and other samples

population		haplotype combination				
HGDP code	name	A1+B6+ C11	A5+B6+ C11	A.other+ B6+C11	B6+C11 total	C11 total
20	Orcadian	16	11	0	27	30
21	Adygei	21	11	0	32	34
22	Russian	26	20	0	46	47
24	Basque	26	17	1	44	48
25	French	19	31	1	51	55
27	Italian	15	6	2	23	24
28	Sardinian	17	33	1	51	55
29	Tuscan	8	5	1	14	16
34	Mozabite	20	24	0	44	50
36	Bedouin	33	49	2	84	88
37	Druze	24	48	6	78	84
38	Palestinian	30	50	4	84	90
50	Balochi	18	19	1	38	45
51	Brahui	24	22	1	47	49
52	Burusho	23	26	0	49	49
54	Hazara	23	6	0	29	29
56	Kalash	40	6	0	46	46
57	Makrani	23	17	0	40	43
58	Pathan (Pashtun)	18	21	1	40	41
59	Sindhi	20	17	1	38	39
86	Maya	1	3	0	4	4
430	BantuSouthAfrica	1	0	0	1	1
464	Mandenka	1	3	0	4	5
465	Yoruba	0	1	0	1	1
494	San	1	0	0	1	1
601	Han (a)	1	0	1	2	3
606	Dai (a)	0	1	0	1	1
607	Daur (a)	2	0	0	2	2
613	Oroqen (a)	3	0	0	3	3
617	Tu (a)	1	0	0	1	1
618	Xibo (a)	3	0	0	3	4
619	Yi (a)	1	0	0	1	1
622	Mongola (a)	3	0	0	3	3
629	Uygur	3	5	0	8	9
677	Cambodian (a)	0	1	0	1	1
699	Yakut	10	2	0	12	13
	Yemen	6	5	0	11	13
	Egypt	10	8	2	20	21
	Ethiopian Jews	3	6	0	9	12
	Ethiopian	7	11	0	18	21
	Saudi	21	15	1	37	37
	Morocco	5	10	1	16	18
	South Indian (mixed)	5	9	0	14	15
**East Asian subtotal (a)**		**14**	**2**	**1**	**18**	**20**
**total**		**532**	**519**	**27**	**1078**	**1152**
