# Supplementary material for: Molecular Phylogeography of a Human Autosomal Skin Color Locus Under Natural Selection
Source: G3 (Bethesda). 2013 Nov 1;3(11):2059–67. doi: 10.1534/g3.113.007484 (PMC3815065; doi:10.1534/g3.113.007484)
Supplement: Supporting Information [file supp_g3.113.007484_TableS15.pdf]

Table S15 Distribution of nucleotide substitutions in C and D regions

nucleotide change	fraction of total	
	hu:chimp (n=1227)	C11D4 (n=269)
A:G or G:A	0.306	0.342
T:C or C:T	0.355	0.346
A:T or T:A	0.077	0.056
C:G or G:C	0.082	0.063
A:C or C:A	0.103	0.097
T:G or G:T	0.077	0.097
